# DeepFold-PLM: accelerating protein structure prediction via efficient homology search using protein language models

**DOI:** 10.1093/bioinformatics/btaf579

**Published:** 2025-10-17

**Authors:** Minsoo Kim, Hanjin Bae, Gyeongpil Jo, Kunwoo Kim, Sung Jong Lee, Jejoong Yoo, Keehyoung Joo

**Affiliations:** Department of Physics, Sungkyunkwan University, Suwon 16419, Korea; Department of Physics, Sungkyunkwan University, Suwon 16419, Korea; Department of Physics, Sungkyunkwan University, Suwon 16419, Korea; Department of Physics, Sungkyunkwan University, Suwon 16419, Korea; Basic Science Research Institute, Changwon National University, Changwon 51140, Korea; School of Computational Sciences, Korea Institute for Advanced Study, Seoul 02455, Korea; Center for Advanced Computation, Korea Institute for Advanced Study, Seoul 02455, Korea

## Abstract

**Motivation:**

Protein structure prediction has been revolutionized and generalized with the advent of cutting-edge AI methods such as AlphaFold, but reliance on computationally intensive multiple sequence alignments (MSA) remains a major limitation.

**Results:**

We introduce DeepFold-PLM, a novel framework that integrates advanced protein language models with vector embedding databases to enhance ultra-fast MSA construction, remote homology detection, and protein structure prediction. DeepFold-PLM utilizes high-dimensional embeddings and contrastive learning, significantly accelerate MSA generation, achieving 47 times faster than standard methods, while maintaining prediction accuracy comparable to AlphaFold. In addition, it enhances structure prediction by extending modeling capabilities to multimeric protein complexes, provides a scalable PyTorch-based implementation for efficient large-scale prediction. Our method also effectively increases sequence diversity ($N_{\text{eff}}$ = 8.65 versus 4.83 with JackHMMER) enriching coevolutionary information critical for accurate structure prediction. DeepFold-PLM thus represents a versatile and practical resource that enables high-throughput applications in computational structural biology.

**Availability and implementation:**

Source codes and user-friendly Python API of all modules of DeepFold-PLM publicly available at https://github.com/DeepFoldProtein/DeepFold-PLM.

## 1 Introduction

Protein structure prediction has experienced a paradigm shift with the advent of AI-driven models such as AlphaFold (Jumper *et al.* 2021, Abramson *et al.* 2024) and RoseTTAFold (Baek *et al.* 2021). These models have achieved near-experimental accuracy, significantly advancing structural biology and biomedical research. The official open-source release of AlphaFold has enabled researchers to optimize prediction pipelines and extend its applications to protein complex modeling, protein design, protein-peptide interactions, protein-ligand interactions, structural validations, ensemble generation, etc. (Anishchenko *et al.* 2021, Bryant *et al.* 2022, Tsaban *et al.* 2022, van Breugel *et al.* 2022, Wong *et al.* 2022, Burke *et al.* 2023, Volk *et al.* 2023). Tools such as ColabFold (Mirdita *et al.* 2022) and OpenFold (Ahdritz *et al.* 2024) have further improved usability and computational efficiency, leading to the widespread adoption of AI-driven structure prediction.

Despite these advances, AlphaFold’s reliance on multiple sequence alignments (MSAs) remains a major limitation. MSAs, which enrich the informational content of protein sequences, play a critical role in the capture of evolutionary covariations that define structural constraints (Baek *et al.* 2021, Jumper *et al.* 2021, Abramson *et al.* 2024). However, generating MSAs is computationally intensive, and conventional MSA search methods often fail to identify remote homologous sequences, particularly for orphan proteins with sparse evolutionary context (Rost 1999, Eddy 2004, Söding 2005, Gabaldón 2008). These limitations pose significant challenges in terms of computational efficiency and scalability, especially in high-throughput applications or when dealing with highly divergent sequences (Eddy 2011, Remmert *et al.* 2011, Buchfink *et al.* 2015, Steinegger and Söding 2017).

Recently, protein structure prediction methods using protein language models (PLMs) (Elnaggar *et al.* 2021, 2023, Rives *et al.* 2021, Lin *et al.* 2022, 2023, Wu *et al.* 2022, Fang *et al.* 2023, Lee *et al.* 2023, Chen *et al.* 2024) have emerged as a promising alternative to MSA-based methods. For example, models such as ESMFold (Lin *et al.* 2023) and OmegaFold (Wu *et al.* 2022) utilize PLMs to encode a query sequence into high-dimensional embeddings that implicitly capture structural and evolutionary features. Although these PLM-based single-query approaches enable rapid predictions by eliminating the need for computationally expensive MSAs, they often fail to capture coevolutionary interactions, which are critical for the accurate prediction of protein structure achieved by MSA-based methods (Chen *et al.* 2024). Beyond structure prediction, PLMs have been rapidly applied across diverse areas of protein research, including sequence search, alignment, design, and functional evaluation (Anishchenko *et al.* 2021, van Breugel *et al.* 2022, McWhite *et al.* 2023, Hamamsy *et al.* 2024, Liu *et al.* 2024, Pantolini *et al.* 2024, Hong *et al.* 2025). For example, PLMSearch (Liu *et al.* 2024) demonstrated up to a threefold increase in sensitivity over MMseqs2 (Steinegger and Söding 2017) in remote homology search while maintaining comparable speed on the SCOPe40-test benchmark.

To address the challenges of efficient MSA generation, we developed DeepFold-PLM, a novel framework that leverages PLMs (Elnaggar *et al.* 2021, 2023, Rives *et al.* 2021, Lin *et al.* 2022, 2023, Wu *et al.* 2022, Fang *et al.* 2023, Lee *et al.* 2023, Chen *et al.* 2024) to construct MSAs rapidly and effectively, with the primary aim of improving the accuracy of downstream structure prediction rather than alignment quality *per se*. Our contributions include: (i) PLM-based remote homology detection and ultra-fast MSA generation (plmMSA), achieved through a newly designed pipeline that delivers 47-fold and 23-fold faster MSA construction compared with AlphaFold’s standard JackHMMER-based pipeline (Eddy 2011) and MMseqs2 (Steinegger and Söding 2017), respectively; (ii) extension of DeepFold (Lee *et al.* 2023) beyond monomer modeling to protein complex prediction; and (iii) complete reimplementation of the pipeline in a scalable PyTorch framework, incorporating model and data parallelism together with advanced computing strategies to efficiently process large proteins and complexes.

We benchmarked DeepFold-PLM on the CASP15 dataset and a diverse set of protein complexes, demonstrating high-speed and reliable protein structure prediction while maintaining prediction accuracy comparable to AlphaFold. Our contrastive learning-based plmMSA method significantly accelerates MSA construction (∼47 times faster than JackHMMER), enhances sequence diversity ($N_{\text{eff}}$ = 8.65 versus 4.83), and improves remote homology detection, especially beneficial for orphan and highly divergent proteins (Gao *et al.* 2022b, Heinzinger *et al.* 2022, Hong *et al.* 2025). In addition, it facilitates rapid and reliable modeling of protein complex interactions.

Overall, DeepFold-PLM serves as a versatile and open source framework that is well suited for developers in computational structural biology, offering high-speed and scalable protein structure prediction.

## 2 Materials and methods

### 2.1 Monomer structure prediction pipeline

**Figure 1.**
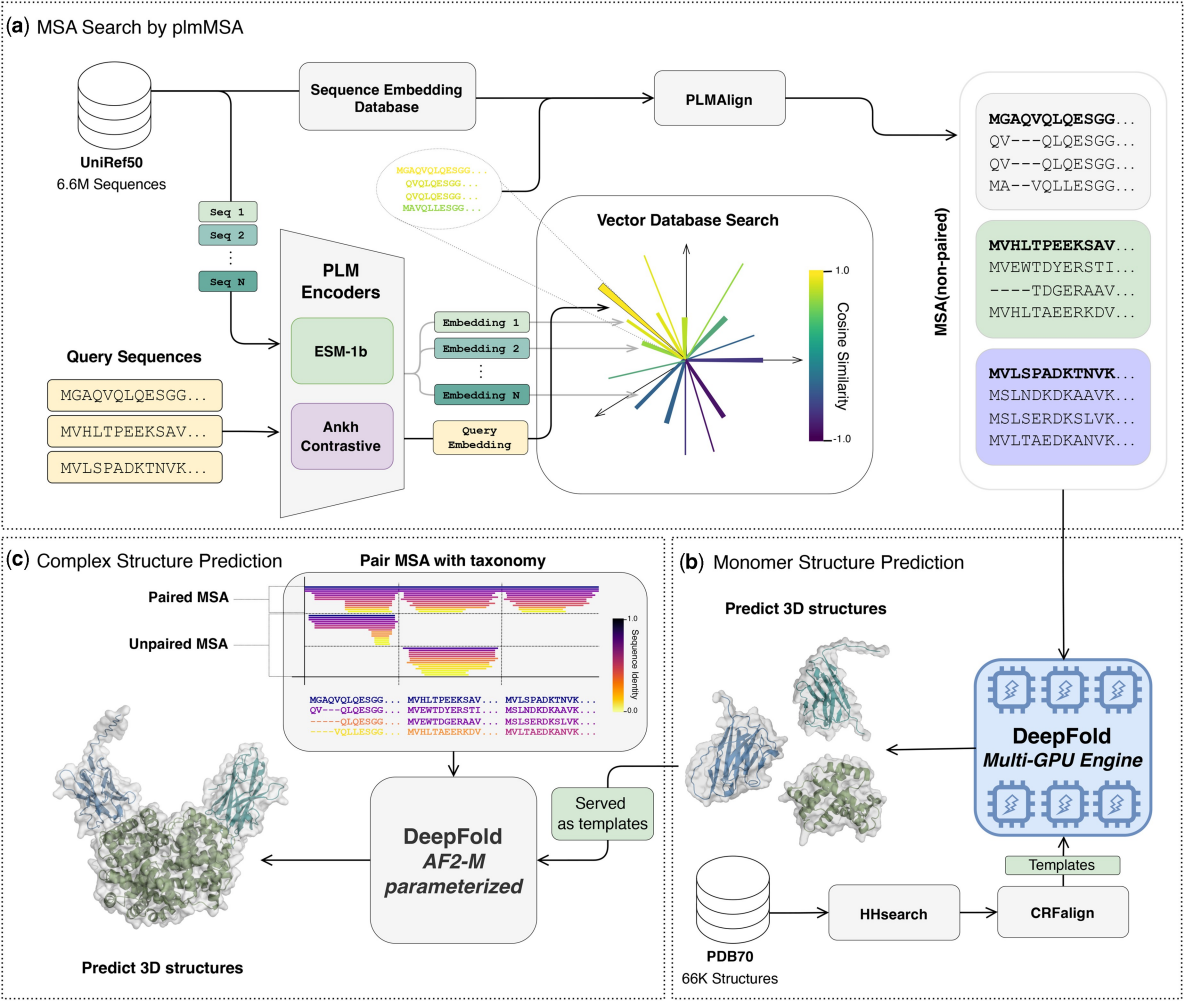
Architecture of DeepFold-PLM pipeline. (a) The plmMSA module constructs MSA using PLM-based alignment. Protein sequences are transformed into dense vector representations through pre-trained PLMs, enabling rapid retrieval of homologous sequences. PLMAlign (Liu *et al.* 2024) then constructs MSAs using vector database embeddings, precomputed ProtT5 embeddings, and query sequence embeddings. (b) The monomer structure prediction module integrates plmMSA-derived constraints and homologous templates from PDB70 to predict 3D protein structures. (c) The complex structure prediction module extends the approach to multimeric protein through template search and coevolutionary analysis. Note that both the monomer and complex structure prediction modules have been fully reimplemented in a scalable PyTorch framework that supports multi-GPU environments, incorporating model and data parallelism together with advanced computing strategies.

The monomer structure prediction pipeline integrates the outputs from the plmMSA module (see Fig. 1a) to predict the 3D structure of monomeric proteins (Fig. 1b). The pipeline operates as follows:

1. **MSA generation**: Initially, the query protein sequence is encoded into a high-dimensional vector embedding using pre-trained protein language models (PLMs), specifically the Ankh Contrastive and ESM-1b encoders. These embeddings facilitate rapid retrieval of homologous sequences from the UniRef50 database through vector similarity searches. PLMAlign then constructs a multiple sequence alignment (MSA) by aligning these sequences based on their embedding vectors.
2. **Template utilization**: Structural templates from the PDB70 database are selected using HHsearch (Steinegger *et al.* 2019), providing spatial constraints to refine predicted structures, especially in conserved domains (Fig. 1b).
3. **Structure prediction**: The resulting MSA and templates are fed into the DeepFold network, which integrates MSA-derived evolutionary information derived from the MSA and spatial constraints from the templates into structural modeling.
4. **Recycling iterations and optimization**: Structures are iteratively refined until convergence, determined by minimal RMSD changes (cutoff at 1 Å).

### 2.2 Choice of PLM models

For sequence embeddings, we used two complementary PLM encoders: the Ankh (Elnaggar *et al.* 2023) and ESM-1b (Rives *et al.* 2021). We selected ESM-1b as a reasonable and representative choice because it is a widely used baseline model (Rives *et al.* 2021), and prior benchmarks have reported that the overall performance difference between ESM-1b and ESM2 is minimal (Elnaggar *et al.* 2023). The Ankh encoder was retrained using MSA-aware contrastive learning, explicitly leveraging MSA data to enhance sensitivity in homology detection and evolutionary analysis. Ankh is not constrained by positional embeddings, making it particularly well-suited for MSA Contrastive Learning that requires processing long sequences. In contrast, ESM-1b, trained via masked language modeling, provides robust general-purpose sequence representations without incorporating explicit coevolutionary signals.

### 2.3 Ankh-contrastive model

Starting with the Ankh model in a frozen state as the base, we improved its homology detection capability through contrastive learning using the OpenProteinSet dataset (Ahdritz *et al.* 2023), which provides MSAs for 270 000 sequences. Note that ESM-1b was not retrained due to the limitations of fixed relative positional embeddings, which restrict its ability to effectively learn from extensive MSA data. Note that we did not perform a formal cross-validation to exclude sequences homologous to CASP15 targets from OpenProteinSet prior to training. However, since MSA-aware contrastive learning aims to learn general-purpose sequence representations rather than memorize specific structures, any potential overlap is expected to have only a limited impact on downstream predictions.

Instead of a dense layer with tanh activation, we used a ConvBert (Jiang *et al.* 2021) configured with an input dimension of 1536, 8 attention heads, a hidden dimension of 768 (half of the input), 1 hidden layer, and a kernel size of 7; the tanh activation function was applied. While SimCSE (Gao *et al.* 2022b) applies a dense layer with tanh activation to the classification token, our implementation passes the embedding of the average-pooled sequence through the same adapter. The learning rate was set to $2\times 10^{-6}$ and adjusted using a cosine scheduler; the softmax temperature was 0.05. Training used the Adam optimizer ($\beta_{1}=0.9$, $\beta_{2}=0.98$, $\epsilon =10^{-8}$), 700 warm-up steps, a maximum sequence length of 1024, a global batch size of 64, 12 target sequences per query, and a test ratio of 0.05, over 2 epochs.

**Figure 2.**
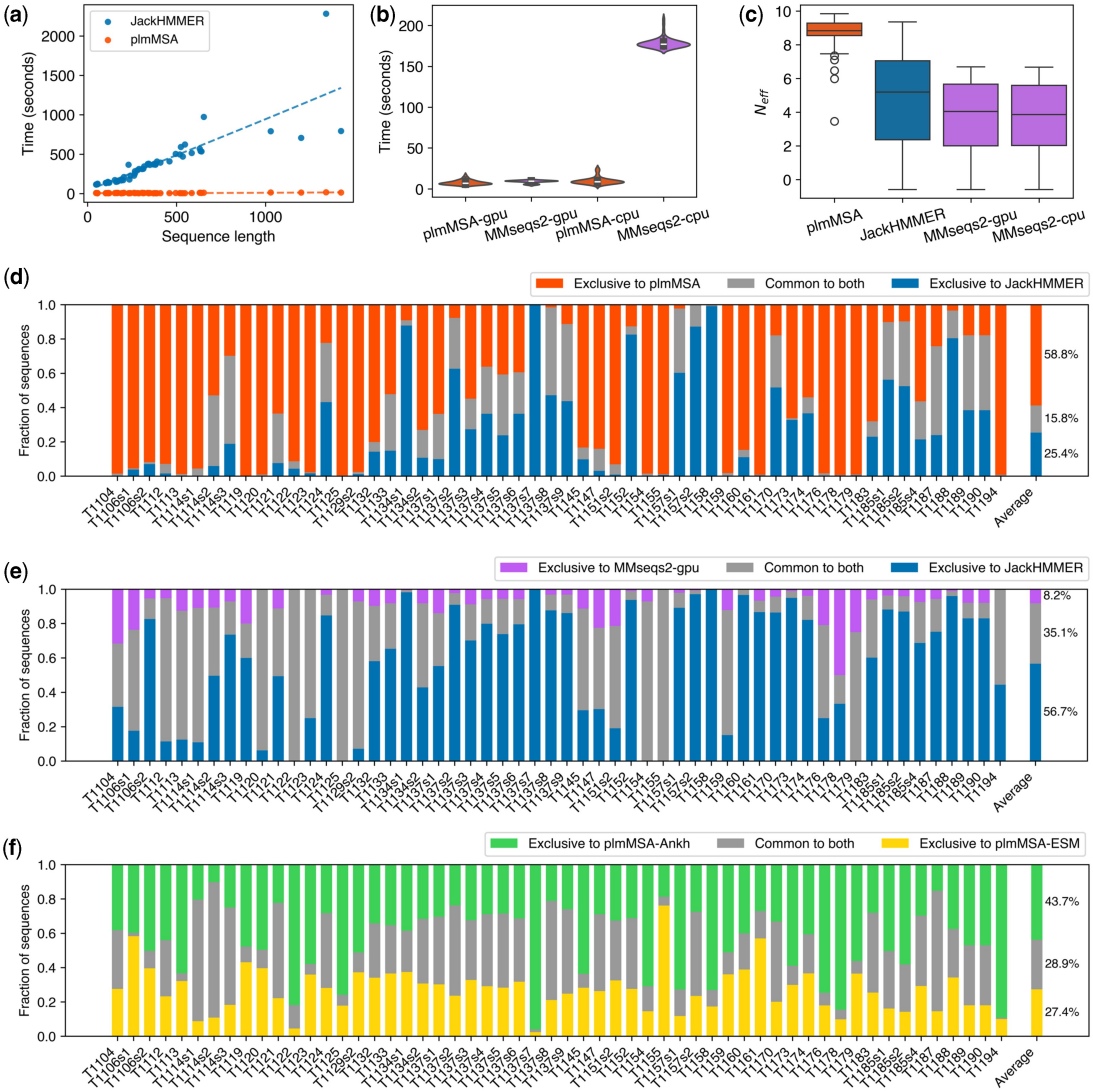
Comparison of plmMSA with other MSA methods on computational speed and sequence diversity across CASP15 targets. (a) Computational time comparison among plmMSA, JackHMMER, MMseqs2-gpu, and MMseqs2-cpu as a function of sequence length. (b, c) Distributions of computation times (b) and effective number of sequences, $N_{\text{eff}}$, (c) across different MSA methods. All MSA computations were performed using identical hardware: dual 24-core Intel Xeon Gold 6442Y processors with 1TB RAM and an NVIDIA RTX 6000 Ada GPU with 48 GB graphics memory. (d) Fractions of sequences exclusively identified by either plmMSA or JackHMMER and those common to both methods across CASP15 targets. (e) Comparison analogous to panel d for JackHMMER and MMseqs2-gpu. (f) Comparison analogous to panel d for plmMSA-Ankh and plmMSA-ESM. Average proportions of exclusive and common sequences™ across all CASP15 targets are indicated on the right side of each panel.

The MSA-aware contrastive learning strategy utilizes a loss function designed to capture the relationships between homologous sequences within multiple sequence alignments. The model produces embeddings that preserve the evolutionary relationships between sequences (Fig. 2, available as supplementary data at *Bioinformatics* online). Given an input tensor $h\in R^{N_{q}\times N_{t}\times H}$ where $N_{q}$ represents the number of query sequences, $N_{t}$ denotes the number of target sequences per query, and *H* is the hidden dimension size, we define the query vector $h_{i1}$ as the first element of *i*th query sequence. This representation serves as the anchor point for our contrastive learning framework. We measure the similarity of two representation vectors as


(1) $$\text{sim}(h_{1},h_{2})=\frac{h_{1}^{⊤}h_{2}}{∥h_{1}∥∥h_{2}∥}$$

where ∥·∥ denotes the L2 norm of the vector. The contrastive loss is then computed as


(2) $$L=\frac{1}{N_{q}}\sum_{i=1}^{N_{q}}L_{i}$$

where


(3) $$L_{i}=-\text{log}\;\frac{\sum_{j=2}^{N_{t}}e^{\text{sim}(h_{i1},h_{ij})/\tau }}{\sum_{j=2}^{N_{t}}e^{\text{sim}(h_{i1},h_{ij})/\tau }+\sum_{j=1}^{N_{t}}\sum_{k=1}^{N_{q}}\left(1-\delta_{ik}\right)e^{\text{sim}(h_{i1},h_{kj})/\tau }}.$$

Note that τ represents the temperature parameter controlling the scaling of the similarity scores.

### 2.4 Vector database of PLM embeddings

For efficiency, we precomputed ESM-1b and Ankh embeddings of all sequences in UniRef50 (63 million entries) (Suzek *et al.* 2015) and stored them in two separate vector databases. For the vector database of Ankh Contrastive encoder, only sequences shorter than 3000 residues were embedded using UniRef50. For the vector database of ESM-1b, due to ESM-1b’s inherent limitation of handling sequences up to 1022 residues, a chunking approach is implemented for longer sequences over 1022 residues.

The vector database implementation uses the FAISS library with the inverted file index (IVF) algorithm (Douze *et al.* 2025) for search speed optimization, clustering database vectors into *K*-distinct centroids during indexing. During search operations, only a subset of clusters is examined based on nearest centroid calculations. For the IVF index, we set the number of centroids (nlist) to 40 000, following the FAISS guideline of nlist  $\approx C\times \sqrt{N}$, where *N* is the database size. Given UniRef50 (N≈63M), a scaling factor of C=5 yields ∼40 000 centroids, providing a practical trade-off between recall and latency. For memory and speed efficiency, we adopted SQ8 (8-bit scalar quantization) because it offers faster encoding/decoding and lower memory overhead compared with product quantization (PQ), which is more computationally demanding. This design choice ensured throughput and memory efficiency for handling ∼63M embeddings.

The required storage for our database is as follows: using average-pooled embeddings *[*1, *D]* for search, Ankh (D=1,536) and ESM-1b (D=1,280) require approximately 91 GB (plus 1.4 GB for metadata) and 80 GB (plus 1.5 GB for metadata), respectively, for a totalling ≈170 GB in the in-memory vector database. Raw full-length embeddings *[L*, *D]* of ProtT5 for PLMalign requires approximately 69−75 TB in disk storage.

### 2.5 MSA construction by plmMSA

**Figure 3.**
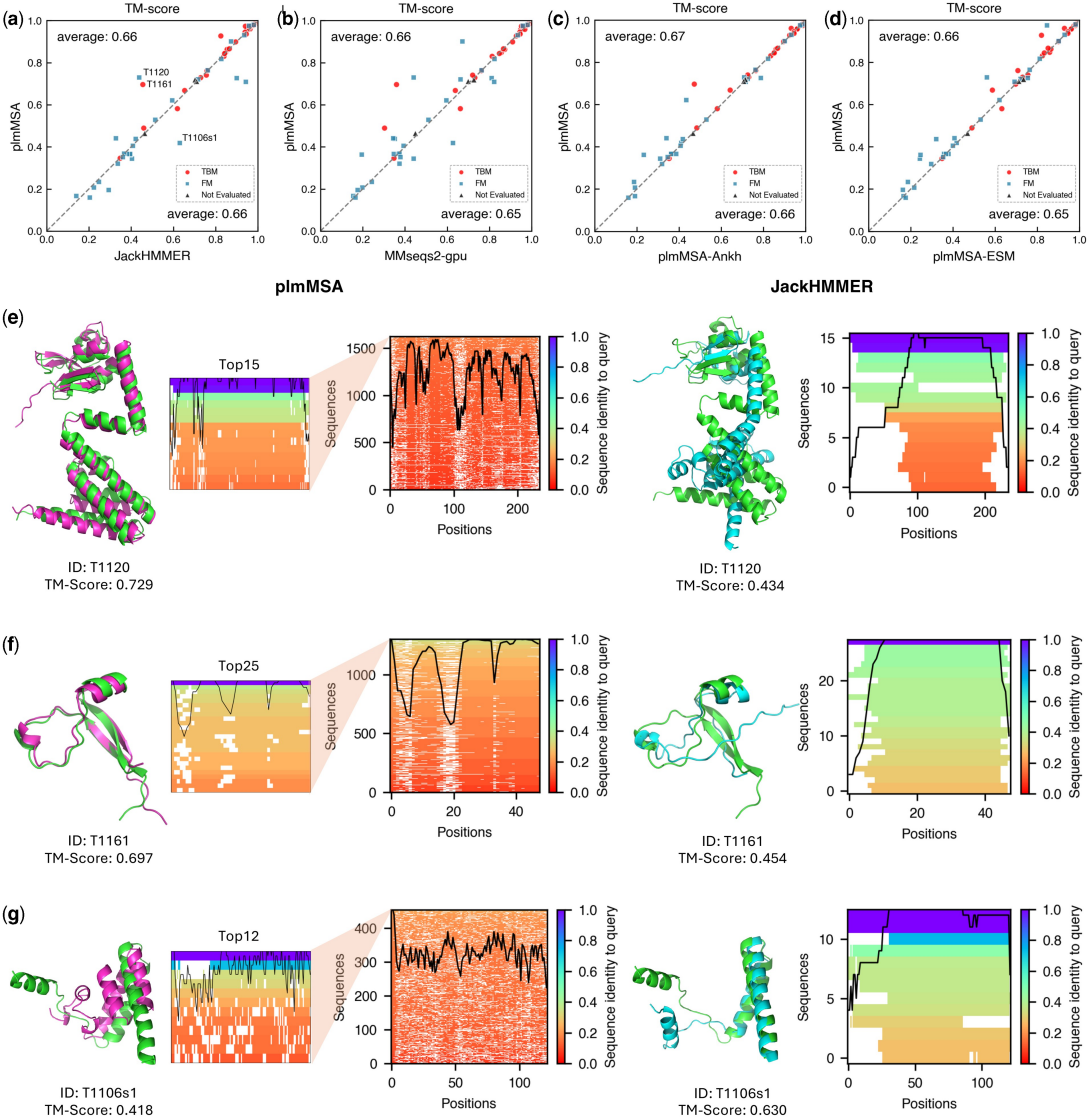
Effects of MSA methods on the structural accuracy of predicted monomeric protein structures. (a–d) Pairwise comparisons of TM-scores for monomer structures predicted using different multiple sequence alignment (MSA) methods: (a) plmMSA versus JackHMMER, (b) plmMSA versus MMseqs2-gpu, (c) plmMSA versus plmMSA-Ankh, and (d) plmMSA versus plmMSA-ESM. Each point represents a single CASP15 target. Points are shaped according to CASP classification: template-based modeling (TBM, circles), free modeling (FM, squares), and targets not evaluated by CASP (triangles). Diagonal lines indicate equal prediction accuracy between the compared methods. TM-scores for individual targets are provided in Tables 2–7, available as supplementary data at *Bioinformatics* online. (e–g) Panels e–g show results of targets T1120, T1161, and T1106s1, respectively. Each panel shows, from left to right: (i) the predicted structure using an MSA generated by plmMSA, (ii) the MSA depth plot from plmMSA, (iii) the predicted structure using an MSA generated by JackHMMER, and (iv) the MSA depth plot from JackHMMER. All predicted structures are overlaid with experimental structures (green) for direct comparison. MSA depth plots illustrate sequence coverage across residue positions, with color-coded sequence identity to indicate alignment quality.

Our plmMSA protocol consists of two key stages: vector database retrieval and PLM-based MSA construction. In the vector database retrieval stage, the query sequence is transformed into a PLM embedding (Ankh Contrastive and ESM-1b encoders). Average pooling is applied to these embeddings. The embeddings are then used to query the vector databases, retrieving the top 1000 matches from each vector database. For sequences shorter than 128 residues, only ESM-1b vector database is utilized for the search process, as it demonstrates superior performance in handling short sequences (Fig. 3, available as supplementary data at *Bioinformatics* online). For longer sequences, both ESM-1b and Ankh vector databases are used. In the PLM-based MSA construction stage, PLMAlign (Liu *et al.* 2024) is used to construct MSA by combining embeddings retrieved from the vector databases and the embedding for the query sequence. Filtering is primarily based on the alignment score, setting the maximum score as the alignment score of the query sequence with itself, and discarding sequences scoring below 20% of the maximum score and with an alignment score <8.0. A detailed breakdown of plmMSA’s computational operations demonstrates its efficiency (Table 8, available as supplementary data at *Bioinformatics* online): vector database search, including query embedding generation, takes an average of 1.21 s, while loading cached embeddings and PLMAlign operations require 3.38 and 3.14 s, respectively. Note that embedding-based alignment (Pantolini *et al.* 2024) can replace PLMalign with comparable performace (Fig. 6, available as supplementary data at *Bioinformatics* online). In contrast, vcMSA (McWhite *et al.* 2023) required >3 h to complete alignment even for the smallest CASP15 target (T1160).

### 2.6 MSA constructions by JackHMMER and MMseqs2

We compared plmMSA with JackHMMER 3.3.2 (Eddy 2011), MMseqs2-gpu (Kallenborn *et al.* 2025), and MMseqs2-cpu (Steinegger and Söding 2017). JackHMMER used the AlphaFold2 inference model with strict multistage filtering using probability cutoffs of $5\times 10^{-4}$, $5\times 10^{-5}$, and $5\times 10^{-7}$, reporting sequences with E-values below $10^{-4}$ and limiting the process to a single iteration. MMseqs2-gpu and MMseqs2-cpu methods utilized default options, with the GPU version incorporating the parameter—index-subset 2 to omit large k-mer data structures and optimize GPU memory usage.

### 2.7 Hardware specifications for the performance comparisons among MSA methods

All computational benchmarks and performance comparisons were conducted using a standardized hardware environment to ensure consistent evaluation across MSA methods: JackHMMER, MMseqs2-cpu, MMseqs2-gpu, and plmMSA. Specifically, computations were performed on a system equipped with dual Intel Xeon Gold 6442Y processors (2.60 GHz, 24 cores per CPU), 1 TB DDR4 RAM, and an NVIDIA RTX 6000 Ada GPU with 48 GB VRAM. All MSA methods were tested under identical hardware conditions to allow fair assessment of computational speed and scalability.

### 2.8 RMSD-based early stopping for optimized predictions

Due to our observation, when the structure seems to be confident, the RMSD of the structure is saturated, and the recycling procedure does not renew the predicted structure. Therefore, when the RMSD of CA atoms between recycled structures is smaller than the cutoff, then the recycling is stopped. The cutoff value is 1 Å adopted from AF2Complex (Gao *et al.* 2022a).

### 2.9 Optimizing memory usage with in-place operations

To reduce memory overhead during inference, in-place operations were implemented, replacing traditional element-wise tensor computations. By discarding intermediate activations unnecessary for the forward pass, the memory footprint is significantly reduced without compromising accuracy. This refactoring ensures efficient use of resources while maintaining compatibility with PyTorch’s flexible and extensible architecture.

### 2.10 Custom CUDA kernels for optimized attention modules

Custom CUDA kernels were developed to optimize attention modules based on FlashAttention (Dao *et al.* 2022), incorporating support for AlphaFold 2-specific bias terms. This approach minimizes memory consumption by allocating only a single copy of the quadratic attention logit tensor, achieving a peak memory reduction of six times less compared to the standard PyTorch implementation.

### 2.11 Improving scalability with dynamic axial parallelism

We adopted dynamic axial parallelism, a feature of FastFold (Cheng *et al.* 2023), to shardedly allocate intermediate MSA and pair representations across GPUs. This approach reduces memory usage and enables efficient all-to-all communication for transposing tensors between GPUs. While communication overhead slightly increases, the overall computation efficiency improves, resulting in faster inference times.

### 2.12 Enhancing throughput with asynchronous chunk broadcasting

To further enhance scalability, asynchronous chunk broadcasting was implemented. AlphaFold 2 suppresses memory costs during inference through a method called chunking, which splits input tensors into chunks, module-specific sub-batch dimensions, and then subsequently processes those modules sequentially for each chunk. This technique is also applied to model parallelism with the pipeline optimization. By processing chunk computation and broadcasting asynchronously, communication latency is effectively masked by ongoing computation, enhancing the pipeline’s overall throughput. When combined with model parallelism, this approach effectively balances memory demands across GPUs.

### 2.13 Multimeric structure prediction pipeline

The multimeric structure prediction pipeline expands upon monomeric predictions by incorporating inter-chain coevolutionary signals using MSA pairing (Fig. 1c). The workflow for multimeric protein structure prediction is outlined below:

1. **Monomer structure prediction**: For uniq input sequences, the monomer structure prediction pipeline generates MSAs and monomer structures.
2. **MSA pairing**: Initially, monomer MSAs generated through the plmMSA module are paired based on taxonomy identifiers from UniProt, enabling biologically meaningful interactions to be captured between interacting protein chains. Unpaired sequences are structured into a block-diagonal matrix, ensuring alignment specificity between chains.
3. **Template integration**: Predicted monomer structures with high per-residue predicted local distance difference test (pLDDT) scores (>70) serve as structural templates.
4. **Multimeric structure prediction**: DeepFold-PLM predicts multimeric structures using AlphaFold-Multimer v2.3 parameters, effectively incoporating MSAs and structural templates to capture inter-chain mutational covariation signals. Similar to monomer prediction, iterative recycling refines complex structures, until convergence, determined by minimal alpha-carbon RMSD (cutoff set at 1 Å).

## 3 Results and discussion

DeepFold-PLM is a protein structure prediction pipeline composed of three core modules: the plmMSA module (Fig. 1a), the monomer structure prediction module (Fig. 1b), and the complex structure prediction module (Fig. 1c). The pipeline begins with the plmMSA module (Fig. 1a), which utilizes pre-trained PLMs to encode protein sequences into high-dimensional embedding vectors capturing evolutionary, structural, and biochemical features. These embeddings facilitate the efficient retrieval of diverse homologous sequences, thus enabling the rapid construction of high-quality MSAs. Next, the monomer structure prediction module (Fig. 1b) leverages homologous sequences and MSA-derived constraints to generate accurate 3D protein structures, incorporating structural information inferred from evolutionary relationships. Finally, the complex structure prediction module (Fig. 1c) builds multimeric protein assemblies by integrating template-based modeling and coevolutionary analysis, capturing inter-subunit interactions to predict biologically relevant complexes.

### 3.1 The plmMSA module for the construction of multiple sequence alignments

#### 3.1.1 Architecture of plmMSA module

The plmMSA module uses two complementary PLM encoders, Ankh (Elnaggar *et al.* 2023) and ESM-1b (Rives *et al.* 2021), which transform sequences into high-dimensional vector embeddings that capture biochemical, structural, and evolutionary properties. We implemented homology detection capabilities into the Ankh encoder through MSA-aware contrastive learning, resulting in the Ankh contrastive encoder. The contrastive learning specifically optimizes the embedding space by clustering homologous sequences closer together while separating non-homologous sequences. The training dataset was derived from OpenProteinSet (Ahdritz *et al.* 2023), including MSAs for approximately 270 000 sequences. In particular, we did not apply contrastive learning to the ESM-1b encoder due to its intrinsic limitation on sequence length (≤1022 residues) (Rives *et al.* 2021), which restricts its effective use of extensive MSA data (Ahdritz *et al.* 2023). Instead, for ESM-1b, we used Ankh developed by Liu *et al.* (2024)

Using the ESM-1b and Ankh contrastive encoders, we precomputed encodings of all sequences in the UniRef50 dataset (Suzek *et al.* 2015). To enable efficient and scalable retrieval, we store these precomputed embeddings in a vector database (Douze *et al.* 2025), allowing rapid similarity-based searches. Homology detection operates through vector comparisons using simple similarity ranking metrics [Equation (1) in the Section 2], significantly reducing computational overhead and enabling near-instantaneous retrieval—requiring only a few seconds of GPU computation per query. Once homologous sequences are identified, PLMAlign (Liu *et al.* 2024) performs filtering and alignments to generate an MSA, leveraging precomputed sequence embedding database for rapid alignment.

Hereinafter, “plmMSA” denotes our PLM-based MSA method combining both Ankh contrastive and ESM-1b encoders, whereas “plmMSA-Ankh” and “plmMSA-ESM” refer to the methods using only Ankh contrastive or ESM-1b encoders, respectively.

#### 3.1.2 Comparison of plmMSA with other MSA methods

Our plmMSA method demonstrates significant improvements in computational speed for the construction of MSA. When evaluated on the CASP15 dataset of 56 monomer structures (Alexander *et al.* 2023) (Table 1, available as supplementary data at *Bioinformatics* online), plmMSA processes queries in an average of 8 s (Table 8, available as supplementary data at *Bioinformatics* online) compared with 365 s for JackHMMER (Eddy 2011) (Fig. 2a) and 179 s for MMseqs2 (Steinegger and Söding 2017) (Fig. 2b), corresponding to 47- and 23-fold speedups, respectively. Unlike JackHMMER, whose runtime increases monotonically with sequence length, plmMSA maintained nearly constant processing times across different sequence lengths, demonstrating excellent scalability (Fig. 2a). Moreover, plmMSA achieved an average effective number of sequences ($N_{\text{eff}}$) of 8.7, compared with 4.8 for JackHMMER and 3.6 for MMseqs2 (Fig. 2c), suggesting that plmMSA is not only computationally efficient but also capable of exploring sequence space not reached by conventional methods. For the benefit of readers, we also benchmarked the experimental MMseqs2-gpu implementation (Kallenborn *et al.* 2025) and found that is was 13% slower than plmMSA (Fig. 2b). Importantly, plmMSA maintains essentially the same speed on both CPU and GPU, whereas MMseqs2 shows a dramatic disparity between CPU and GPU performance (Fig. 2b), underscoring the architectural efficiency and practical versatility of plmMSA.

A detailed comparative analysis between plmMSA and JackHMMER further revealed that plmMSA identifies a significant number of unique sequences not detected by JackHMMER across CASP15 targets (Fig. 2d). Within the combined superset of sequences identified by both methods, an average fraction of only 15% were common (gray bars, Fig. 2d), while 58% were exclusive to plmMSA (red bars, Fig. 2d). This finding suggests that the fundamentally different principle implemented in plmMSA enables it to effectively explore regions of sequence homology space that are inaccessible to conventional MSA methods. In contrast, sequences retrieved by MMseqs2 largely represented a subset of those obtained by JackHMMER, implying limited complementary exploration beyond JackHMMER’s sequence homology space (Fig. 2e). Specifically, within the combined superset of sequences identified by MMseqs2 and JackHMMER, an average of 92% were either common (gray bars, Fig. 2e) or exclusive to JackHMMER (blue bars, Fig. 2e), with only 8% exclusive to MMseqs2-gpu (purple bars, Fig. 2e).

Figure 2f illustrates the complementary nature of sequence homology searches by Ankh and ESM within the plmMSA framework. An average of 43% and 27% of sequences were exclusively retrieved by plmMSA-Ankh (green bars, Fig. 2f) and plmMSA-ESM (yellow bars, Fig. 2f), respectively, with 28% of sequences common to both encoders (gray bars, Fig. 2f). Notably, plmMSA-Ankh tends to retrieve more unique sequences for most targets, indicating a potentially broader exploration of the sequence similarity space. This advantage likely stems from Ankh’s intrinsic ability to handle sequences without length restrictions, whereas ESM-1b is constrained by fixed sequence length. Consequently, plmMSA-Ankh shows a greater average $N_{\text{eff}}$ of 8.29 compared to 7.75 for plmMSA-ESM (Fig. 2c).

To facilitate practical usage and integration into bioinformatics workflows, we also developed a user-friendly Python API for plmMSA, which is publicly available and easily implementable by researchers (https://github.com/DeepFoldProtein/DeepFold-PLM).

### 3.2 The monomer structure prediction module

#### 3.2.1 Multi-GPU architecture of the structure prediction module

To enable efficient structure prediction across multiple GPUs in DeepFold-PLM, we reimplemented our previous DeepFold code (Lee *et al.* 2023) in PyTorch, incorporating advanced parallelization strategies such as model and data parallelism, dynamic axial parallelism, and asynchronous chunk broadcasting. Importantly, we optimized the attention module, which accounts for the most of computation time and memory usage during structure inference. In addition to these architectural improvements, we retained key features from the original DeepFold, including optimized loss functions—torsion angle, frame-aligned point error (FAPE), and side-chain confidence terms, which collectively improved backbone and side-chain geometry (Lee *et al.* 2023). See Methods for further implementation details.

#### 3.2.2 Accuracy of predicted protein monomer structures using plmMSA

To assess the effect of MSA quality on the structure prediction accuracy, we compared structures predicted using plmMSA- and JackHMMER-generated MSAs across 56 monomer targets from the CASP15 dataset (Fig. 3a). Although structure template features from PDB70 were implemented (Fig. 1b), they were not used in our evaluations, as the primary focus here was on assessing the contribution of MSAs. Note that DeepFold-PLM and AlphaFold2 produce comparable structure predictions when given the same MSA input (Fig. 1, available as supplementary data at *Bioinformatics* online). plmMSA achieves structural prediction accuracy comparable to JackHMMER with both methods attaining an average TM-score of 0.66 (Fig. 3a). Furthermore, comparisons of plmMSA with MMseqs2 variants also show similar performance, with average TM-scores of 0.66 and 0.65, respectively (Fig. 3b and c). Overall, these comparisons confirm that plmMSA efficiently retrieves diverse yet relevant sequences, thereby significantly improving computational speed without compromising the quality of the generated MSAs.

In a detailed comparison between plmMSA and JackHMMER, three targets (T1120, T1161 and T1114s1) demonstrated improved performance with plmMSA relative to JackHMMER, showing TM-score differences >0.22. The enhanced performance in these cases can be attributed to the PLM’s increased sensitivity, which successfully identified additional homologous sequences missed by JackHMMER (see MSA depth plots in Fig. 3e, f for illustrative examples). Conversely, for three other targets (T1106s1, T1145 and T1174), plmMSA underperformed relative to JackHMMER despite retrieving significantly more sequences. This underperformance primarily arose from inconsistent gap patterns within the MSAs (see MSA depth plots for T1106s1 in Fig. 3g), performance discrepancies are predominantly related to challenges in sequence filtering. These findings highlight potential areas for further refinement in the plmMSA approach, particularly regarding improved filtering strategies and gap consistency within alignments.

Among the targets from CASP16, the structures of only 21 targets are publicly available in PDB as of August, 2025 (Table 12, available as supplementary data at *Bioinformatics* online). Our analysis of these targets shows comparable TM-score performance among plmMSA, JackHMMER, and MMseqs2 (Fig. 7, available as supplementary data at *Bioinformatics* online), indicating that the conclusions drawn from CASP15 extend consistently to CASP16.

#### 3.2.3 Complementarity of Ankh and ESM-1b models in structure prediction performance

Comparisons of TM-scores predicted using plmMSA with those obtained using plmMSA-Ankh (Fig. 3d) and plmMSA-ESM (Fig. 3e) revealed that plmMSA significantly improved TM-scores for four and seven targets, respectively, without adversely affecting the TM-scores of other targets. Our analysis shows that these gains arose from the complementary strengths of each model, which are optimized for different sequence lengths.

#### 3.2.4 Computational efficiency and scalability of the structure prediction module

**Figure 4.**
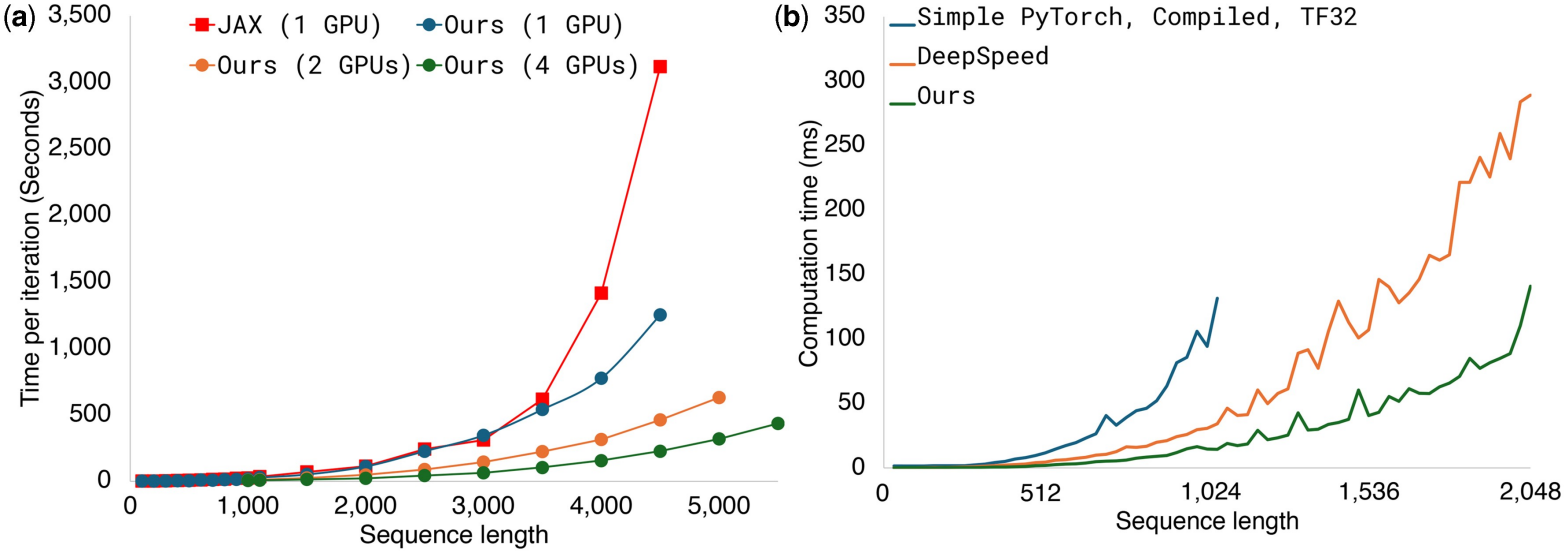
Computational efficiency and multi-GPU scalability of the PyTorch-based DeepFold-PLM. (a) Overall computation time per recycling iteration is shown as a function of protein sequence length. The optimized DeepFold-PLM PyTorch implementation demonstrates near-linear scalability, reducing iteration time with multiple GPUs, while single-GPU performance (NGPUS = 1, circles) is comparable to AlphaFold’s JAX implementation (squares). (b) Computation times of each attention implementation are shown as a function of protein sequence length. Our implementation of biased attention achieves a 6-fold speedup over the standard PyTorch implementation at a sequence length of 1024. The simple PyTorch baseline was compiled using torch.compile with NVIDIA TensorFloat32 (TF32) tensor cores enabled. The DeepSpeed results correspond to the EvoFormerAttention kernel provided by DeepSpeed (Song *et al.* 2023). Benchmarks were conducted using NVIDIA A100 SXM4 GPUs interconnected via NVLink.

Our PyTorch implementation, optimized for multi-GPU architectures, significantly improves the computational performance of protein structure prediction across various GPU configurations (1, 2, and 4 NVIDIA A100 GPUs) (Fig. 4a). On a single GPU, our PyTorch implementation outperforms AlphaFold’s original JAX implementation for sequences longer than 3000 residues (Fig. 4a). This performance gain is primarily attributed to the optimization of the attention module with custom CUDA kernels, yielding a 6-fold speedup over a simple PyTorch baseline and a 2-fold speedup over DeepSpeed’s EvoformerAttention (Fig. 4b). When using multiple GPUs, computational time decreases approximately linearly with GPU count, significantly enhancing throughput. Overall, these technical optimizations enable DeepFold-PLM to efficiently support high-throughput applications and large-scale structural biology research, providing a practical advantage.

### 3.3 Complex structure prediction module

#### 3.3.1 Development of the complex structure prediction module

We developed the complex structure prediction module in DeepFold-PLM (Fig. 1c) by adopting the parameters of AlphaFold-Multimer v2.3 (AF2-Multimer) (Evans *et al.* 2022), while introducing several new features. (i) We introduced a novel feature-generation pipeline that pairs monomer MSAs using taxonomy identifiers from UniProt (Bateman *et al.* 2022) and concatenates unpaired sequences into a block-diagonal MSA matrix with off-diagonal padding. This facilitates the detection of mutational correlations between interacting protein chains (Zhou *et al.* 2018) and enables greater sequence diversity by performing unbiased MSA sampling across recycling iterations. (ii) Instead of performing template searches against the PDB, we reused predicted monomer structures with high per-residue pLDDT scores (>70) as structural templates. This strategy eliminates dependence on HMM-based homology searches and allows the pipeline to operate independently of external sequence or structure databases. Monomer predictions often tend to serve as effective structural guides during the recycling process, steering the model toward consistent multimer configurations. (iii) Early stopping of recycling iteration was introduced to reduce computational overhead by terminating the process once the alpha-carbon distances stabilized, based on RMSD between iterations. (iv) To improve scalability and reduce memory usage, we implemented model parallelism and customized the PyTorch backend. These enhancements enable the pipeline to efficiently handle large multimeric protein complexes, facilitating high-throughput structural prediction.

**Figure 5.**
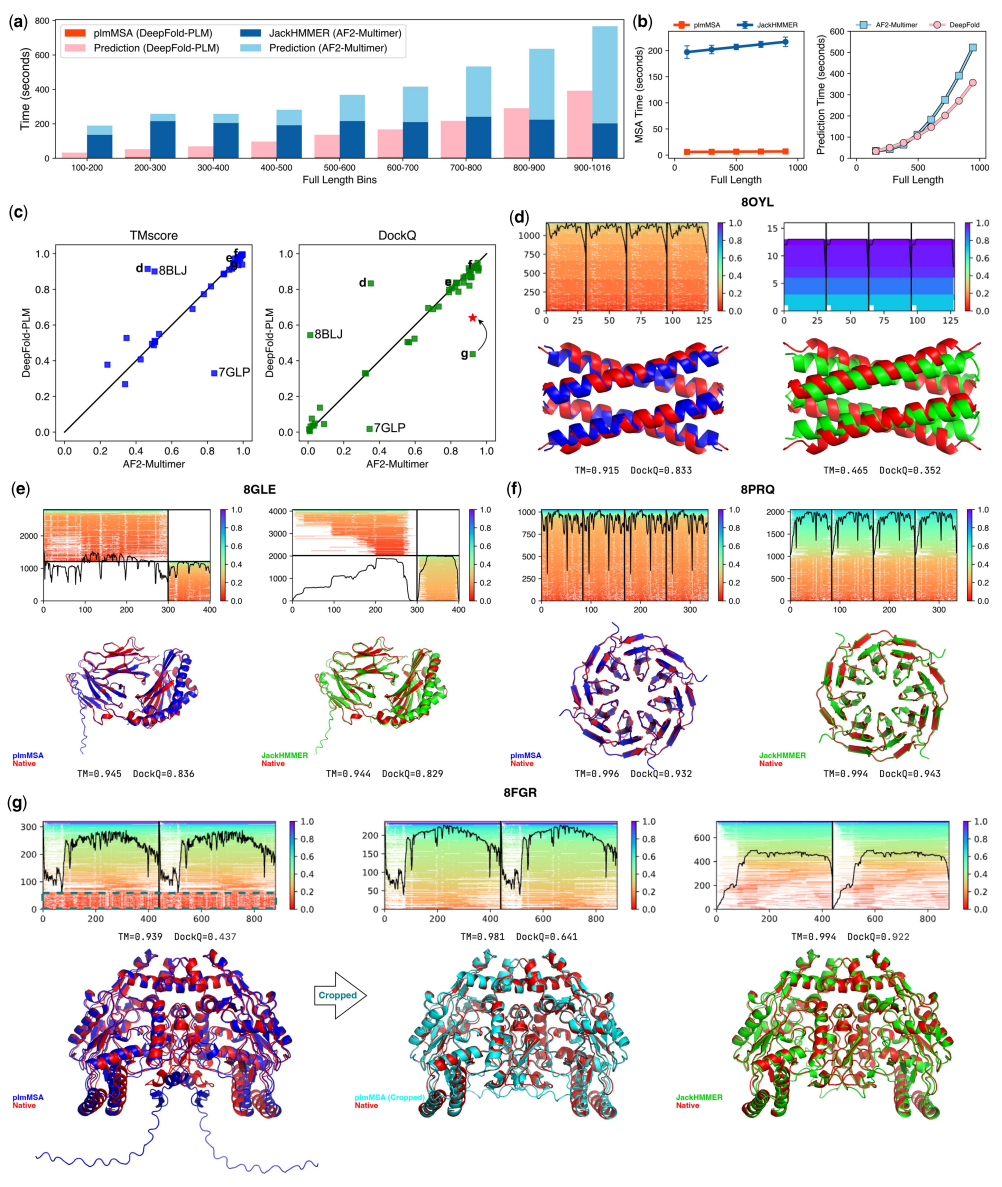
Performance comparison between DeepFold-PLM and AF2-Multimer in protein complex structures prediction. (a) Comparison of end-to-end computational times for DeepFold-PLM and AF2-Multimer across benchmark protein complexes, binned by full sequence length. Each stacked bar represents the cumulative time required for MSA generation (plmMSA for DeepFold-PLM and JackHMMER for AF2-Multimer) and structure prediction. (b) Comparisons of MSA generation times (left) and structure prediction times (right) as a function of sequence length. The color scheme is consistent with panel a. (c) Comparisons of TM-scores (left) and DockQ scores (right) predicted using DeepFold-PLM and AF2-Multimer. The near-diagonal distribution indicates that both methods yield comparable performance across most targets. Data points corresponding to panels d to g are marked next to the symbols. (d–f) Representative successful predictions. Each panel shows (top to bottom, left to right): (i) MSA depth profile from plmMSA; (ii) predicted structure from DeepFold-PLM (blue); (iii) MSA depth profile from JackHMMER; (iv) predicted structure from AF2-Multimer (green). All predictions are aligned to the native structure (red) using US-align (Zhang *et al.* 2022). (d) For 8OYL, plmMSA retrieved a more diverse set of homologous sequences, leading to improved coevolutionary signal and superior accuracy in both global fold and interface geometry (TM = 0.915, DockQ = 0.833) compared to AF2-Multimer (TM = 0.465, DockQ = 0.352). (e, f) For 8GLE and 8PRQ, both pipelines produced similarly accurate structures, as reflected by comparable TM-scores and DockQ values. (g) Representative failure case (8FGR). The initial plmMSA contained noisy sequences (dashed box), leading to poor interface prediction (DockQ = 0.437). Manual cropping of misaligned regions restored accuracy (DockQ = 0.641), highlighting the importance of filtering in high-diversity MSAs.

As shown in Fig. 5a and b, DeepFold-PLM significantly reduces computational time compared to the AF2-Multimer pipeline, particularly in the MSA generation stage. plmMSA offers near-constant runtime across a broad range of sequence lengths, while JackHMMER-based MSA generation in the AF2-Multimer pipeline dominates the total runtime and scales with sequence length. This efficiency is further amplified by DeepFold-PLM’s GPU-optimized PyTorch implementation, which offers improved scalability in the structure prediction stage as sequence length increases. Together, these results demonstrate that DeepFold-PLM is well suited for high-throughput complex structure prediction, especially for large and computationally demanding targets.

#### 3.3.2 Evaluation of the complex structure prediction module

The performance of DeepFold-PLM in predicting protein complex structures was evaluated on 50 diverse protein assemblies deposited in PDB after 30 September 2021, including two challenging cases (7GLP and 8FGR) that were chosen to illustrate the current limitations of our method (Table 9, available as supplementary data at *Bioinformatics* online). For a fair comparison, we used the standard AF2-Multimer protocol with JackHMMER-generated MSAs from the UniRef50 database, the same sequence database used by DeepFold-PLM. As summarized in Fig. 5c, DeepFold-PLM achieves overall predictive accuracy comparable to AF2-Multimer. The TM-score and DockQ scatter plots (Fig. 5c) exhibit a dense near-diagonal distribution, indicating that both pipelines yield similar structural outcomes across most targets. Notably, we observed that for structures with TM-scores below 0.6, the corresponding DockQ values often drop sharply, approaching zero (Zhu *et al.* 2023). This is likely due to the fact that DockQ primarily reflects the quality of the interface between protein subunits, rather than the overall fold of the individual chains. Given that the benchmark set comprises structurally challenging complexes, these results suggest that further improvements in the AF2-Multimer model will be necessary to enhance interface-level accuracy. Figure 5d–g present representative examples in which DeepFold-PLM outperforms (Fig. 5d), matches (Fig. 5e and f), or underperforms (Fig. 5g) AF2-Multimer.

#### 3.3.3 Beneficial impact of sequence diversity on complex prediction

One key advantage of the DeepFold-PLM pipeline lies in its ability to identify a broader and more diverse set of homologous sequences compared to traditional HMM-based methods such as JackHMMER. This increased sequence diversity enables the capture of more informative coevolutionary signals, thereby improving the accuracy of predicted protein complex structures. For example, in the case of 8OYL (Fig. 5d), plmMSA successfully retrieved a substantially wider range of homologous sequences, resulting in a more robust statistical representation of residue-residue covariation. This, in turn, improved the model’s capacity to infer inter-chain contacts and accurately predict interface geometry. The structure predicted by DeepFold-PLM showed markedly better agreement with the native structure (TM-score = 0.912, DockQ = 0.813) compared to that predicted by AF2-Multimer (TM-score = 0.448, DockQ = 0.180), demonstrating the benefit of enriched sequence diversity in multimeric protein modeling.

#### 3.3.4 Limitations and challenges in complex prediction

While the DeepFold-PLM pipeline generally yields complex structure predictions comparable to or better than those based on standard AF2-Multimer procedure, we also identified two failure cases that were selected to highlight recurring challenges of the method. In Fig. 5g, the target 8FGR illustrates a failure case where plmMSA produced a diverse but noisy alignment containing many low-quality sequences. While this resulted in accurate monomer predictions (TM-scores >0.9), the quality of the complex assembly was poor (DockQ = 0.278). After manual removing of misaligned regions, interface accuracy improved substantially (DockQ = 0.647), as indicated by the red asterisk in Fig. 5c. In 7GLP, spurious sequences impaired detection of inter-chain covariation (Fig. 5, available as supplementary data at *Bioinformatics* online). These cases highlight the trade-off of increased sequence diversity in plmMSA: while it enhances contact prediction, it also increases sensitivity to noisy or weakly homologous sequences, underscoring the need for improved filtering strategies and embedding efficiency. Addressing these challenges will be essential to making DeepFold-PLM a more scalable and accurate platform for complex structure prediction, particularly when expanding beyond the limitations of the UniRef50 database.

#### 3.3.5 Template-based enhancement using predicted monomer structures

One representative example of template-based enhancement is the target 8BLJ (Fig. 5c), where the use of predicted monomer structures as templates led to significantly improved complex prediction accuracy, as illustrated in Fig. 4, available as supplementary data at *Bioinformatics* online. In structure prediction pipelines such as AlphaFold-Multimer, the use of monomer models complements sequence-based inputs like MSAs. While MSAs provide evolutionary insights into coevolutionary patterns and residue-residue interactions, monomer templates offer a structural starting point that constrains the search space for assembling complexes. This dual-input strategy leverages both evolutionary and structural signals to improve global topology and interface accuracy. In DeepFold-PLM, incorporating high-confidence monomer predictions as templates was found to enhance both TM-scores and DockQ scores for complex predictions, as shown in Fig. 4, available as supplementary data at *Bioinformatics* online. These templates provide spatial constraints that help align interacting residues and guide subunit positioning, thereby improving structural convergence during recycling.

## 4 Conclusion

We introduced DeepFold-PLM, an advanced computational framework that significantly accelerates protein structure prediction by integrating cutting-edge protein language models with efficient multiple sequence alignment construction. Our plmMSA method substantially improves the speed of sequence homology searches, achieving a remarkable 47-fold acceleration compared to traditional methods like JackHMMER, while maintaining near-constant-time performance independent of sequence length. Importantly, plmMSA can also retrieve homologous sequences that are not detected by conventional tools such as JackHMMER or MMseqs2, thereby uncovering hidden sequence space and enriching evolutionary diversity ($N_{\text{eff}}$= 8.65), which can provide clear benefits in challenging prediction cases. Using high-dimensional vector embeddings and contrastive learning, DeepFold-PLM thus improves computational efficiency while simultaneously enhancing the quality of coevolutionary information critical for accurate structural predictions.

We benchmarked DeepFold-PLM on the CASP15 and CASP16 dataset and a diverse set of protein complexes, demonstrating high-speed and reliable protein structure prediction while maintaining prediction accuracy comparable to AlphaFold. The synergy between Ankh and ESM-1b encoders contributes to robust performance across a wide range of sequence lengths, enhancing the adaptability of DeepFold-PLM for diverse applications in structural biology. Additionally, by fully reimplementing the pipeline in PyTorch, introducing parallelism strategies, and integrating custom CUDA kernels, we provide a scalable and open-source framework that demonstrates excellent performance on multi-GPU systems, enabling large-scale studies requiring massive structure predictions.

Future work will focus on scaling the embedding database, refining sequence filtering methods to mitigate alignment noise, and further improving MSA quality metrics (see Supplementary 4, available as supplementary data at *Bioinformatics* online for additional analysis on MSA quality). By addressing these areas, DeepFold-PLM aims to establish itself as a versatile and indispensable tool for advancing computational structural biology and enabling broader adoption across the scientific community.

## Supplementary Material

btaf579_Supplementary_Data

## Data Availability

We obtained all the protein targets from https://predictioncenter.org/ and structures from https://www.rcsb.org/. For MSA-aware Contrastive Learning for Ankh, we used OpenProteinSet https://registry.opendata.aws/openfold/. For vector database construction, we downloaded UniRef50 from UniProt https://www.uniprot.org/. The parameters for Ankh Contrastive model are available at https://huggingface.co/DeepFoldProtein/Ankh-Large-Contrastive.

## Code availability

Source codes for plmMSA and DeepFold in PyTorch are available under an open-source license at https://github.com/DeepFoldProtein/DeepFold-PLM. We also utilized publicly available software in this work, including AlphaFold 2 (https://github.com/google-deepmind/alphafold), Ankh (https://github.com/agemagician/Ankh), and ESM-1b (https://github.com/facebookresearch/esm). The following software versions were used in this study: FAISS 1.7.2, JackHMMER 3.3.2, MMseqs2.
