# Supplementary material for: DeepFold-PLM: accelerating protein structure prediction via efficient homology search using protein language models
Source: Bioinformatics. 2025 Oct 17;41(11):btaf579. doi: 10.1093/bioinformatics/btaf579 (PMC12598648; doi:10.1093/bioinformatics/btaf579)
Supplement: btaf579_Supplementary_Data [file btaf579_supplementary_data.pdf]

Supplementary Information for: DeepFold-PLM: Accelerating Protein Structure Prediction via Efficient Homology Search Using Protein Language Models

Minsoo Kim[1,*], Hanjin Bae[1,*], Gyeongpil Jo[1],Kunwoo Kim[1], Sung Jong Lee[2], Jejoong Yoo[1,†], Keehyoung Joo[3,†]

[1]Department of Physics, Sungkyunkwan University, Suwon, Korea.
[2]Basic Science Research Institute, Changwon National University, Changwon 51140, Korea.
[3]Center for Advanced Computation, Korea Institute for Advanced Study, Seoul, Korea.
[*]These authors contributed equally to this work.
[†]Corresponding authors: jejoong@skku.edu (J.Y.), newton@kias.re.kr (K.J.)

Contents

S1 Supplementary Methods **3**
S1.1 plmMSA algorithm 3
Algorithm 1. plmMSA Pipeline for MSA Construction 3

S2 Supplementary Figures **4**
Figure S1. Comparison of DeepFold and AlphaFold2 parameters using JackHMMER MSA 4
Figure S2. MSA-aware contrastive learning for homology detection. 5
Figure S3. Performance comparison of plmMSA-Ankh and plmMSA-ESM across short and long protein sequences. 6
Figure S4. Impact of predicted monomer-templates inclusion on prediction accuracy. 7
Figure S5. Alignment depth comparison for failure cases. 8
Figure S6. Performance comparison of PLMAlign and EBA alignments 9
Figure S7. Performance benchmarking of plmMSA on CASP16 targets 10

S3 Supplementary Tables **11**
Table S1. List of CASP15 benchmark targets used for evaluating monomer structure predictions. 11
Table S2. Performance evaluation of JackHMMER on CASP15 targets. 12
Table S3. Performance evaluation of plmMSA on CASP15 targets. 13
Table S4. Performance evaluation of MMseqs2-gpu on CASP15 targets. 14
Table S5. Performance evaluation of MMseqs2-cpu on CASP15 targets. 15
Table S6. Performance evaluation of plmMSA-Ankh on CASP15 targets. 16

Table S7. Performance evaluation of plmMSA-ESM on CASP15 targets. . . . 17
Table S8. Detailed execution time analysis of the plmMSA module for CASP15 targets. 18
Table S9. Performance evaluation on protein complex targets. 19
Table S10. End-to-end computational times across target complexes. 20
Table S11. Performance evaluation of EBA on CASP15 targets. 21
Table S12. Performance evaluation of MSA generation methods on CASP16 targets 22

S4 Evaluating MSA quality for structural accuracy **24**

S1 Supplementary Methods

S1.1 plmMSA algorithm

The plmMSA algorithm constructs multiple sequence alignments using protein language models. It combines ESM-1b and Ankh Contrastive model embeddings to retrieve homologous sequences and generate alignments for structure prediction. The algorithm filters the results using alignment scores derived from PLMAlign.

Algorithm 1 plmMSA Pipeline for MSA Construction

Require: Query protein sequence Q
Ensure: Multiple Sequence Alignment (MSA) for Q

// **1. Query Embedding Generation**
if length(Q) $<$ 128 **then**
    $E_Q \leftarrow$ ESM-1b(Q)
    $E_Q \leftarrow$ AvgPool(E_Q)
else
    $E_Q^{(A)} \leftarrow$ Ankh Contrastive(Q)
    $E_Q^{(A)} \leftarrow$ AvgPool($E_Q^{(A)}$)
    $E_Q^{(E)} \leftarrow$ ESM-1b(Q)
    $E_Q^{(E)} \leftarrow$ AvgPool($E_Q^{(E)}$)
end if
// **2. Retrieve Homologous Sequences**
if length(Q) $<$ 128 **then**
    $C \leftarrow$ Retrieve(E_Q, ESM-1b VDB, N)
else
    $C_A \leftarrow$ Retrieve($E_Q^{(A)}$, Ankh Contrastive VDB, N)
    $C_E \leftarrow$ Retrieve($E_Q^{(E)}$, ESM-1b VDB, N)
    $C \leftarrow C_A \cup C_E$
end if
// **3. Load Candidate Embeddings from Sequence Embedding Database**
for each $s \in C$ **do**
    $E_s \leftarrow$ LoadEmbed(s, SeqEmbed DB)
end for
// **4. Initial Alignment and Score Computation**
$MSA_{\text{init}} \leftarrow$ PLMAlign(Q, C, $\{E_s\}_{s \in C}$)
// **5. Filter Candidates Based on Alignment Scores**
Let $S_{\text{self}} \leftarrow$ PLMAlign(Q, Q) {/* S_{self} is the alignment score of Q with itself */}
Define threshold $T \leftarrow \min(0.2 \times S_{\text{self}}, 8.0)$
$C_{\text{filt}} \leftarrow \{\, s \in C \mid \text{Score}(s) \geq T \,\}$
// **6. Final MSA Construction**
$MSA \leftarrow \{\, s \in MSA_{\text{init}} \mid s \in C_{\text{filt}} \,\}$
return MSA

S2 Supplementary Figures

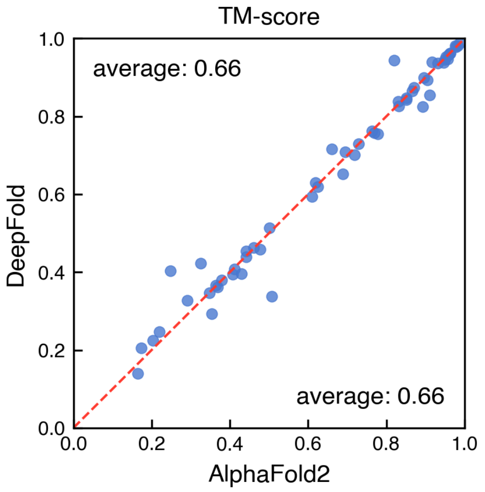

Figure S1: Comparison of DeepFold and AlphaFold2 performance on CASP15 targets using the same JackHMMER MSAs.

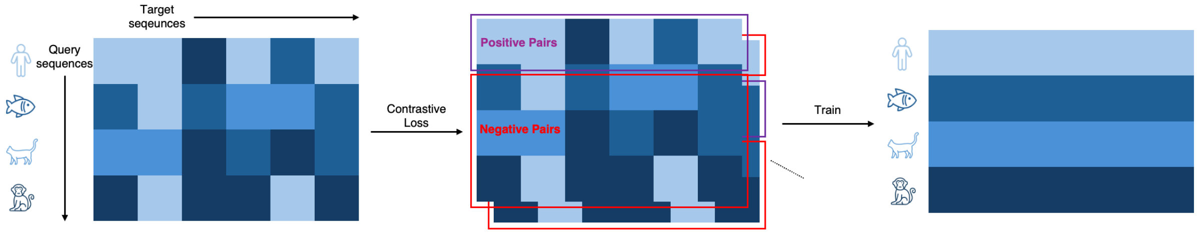

Figure S2: **MSA-aware contrastive learning for homology detection.** The pre-trained model takes a query sequence and a set of target sequences from an MSA, where homology relationships are initially unknown. MSA-aware contrastive loss defines aligned target sequences as positive pairs with the query, while treating all other sequences as negatives. This encourages homologous pairs to be mapped closer together in the embedding space, while non-homologous pairs are pushed farther apart. Each tile represents an individual sequence, with sequences in the same row sampled from the same MSA.

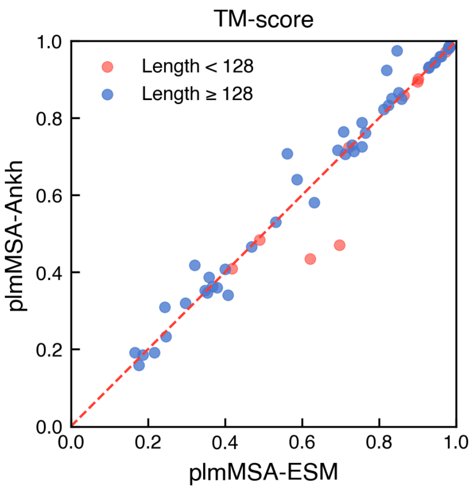

Figure S3: **Comparison of plmMSA-Ankh and plmMSA-ESM performance across sequence length categories.** The figure shows TM-scores for sequences shorter and longer than 128 residues, demonstrating that plmMSA-ESM outperforms plmMSA-Ankh in the short-sequence regime. This observation supports the selective use of the ESM-1b vector database for short sequences, as described in the main text.

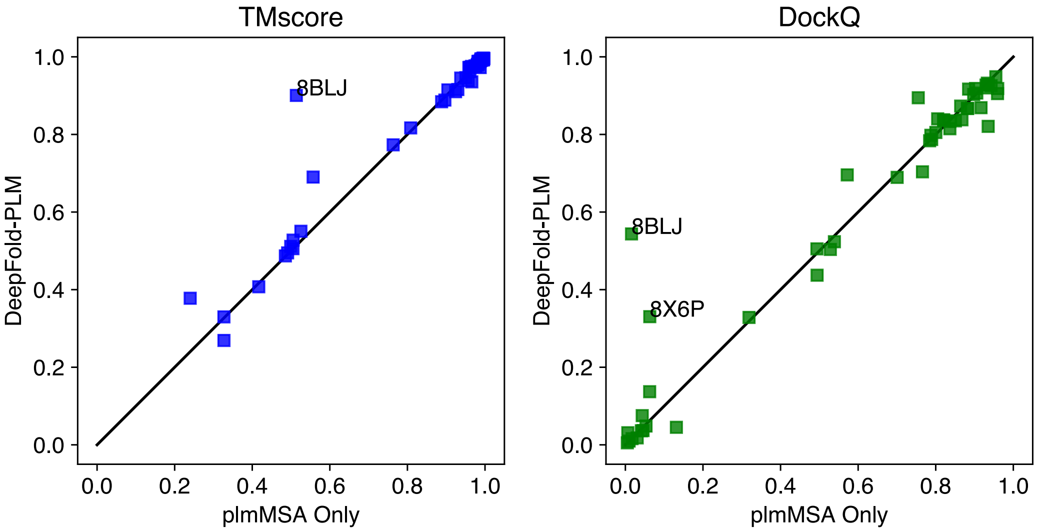

Figure S4: **Effect of adding predicted monomer structures as templates in the plmMSA inference pipeline.** (Left) Scatter plot of the TM-score obtained for each complex with plmMSA alone (x-axis) versus plmMSA augmented with monomer templates (y-axis). (Right) Equivalent comparison for the DockQ score. The diagonal marks parity; points above the line represent complexes whose accuracy improves when monomer templates are supplied. Most complexes show systematic gains in both global topology (TM-score) and interface quality (DockQ).

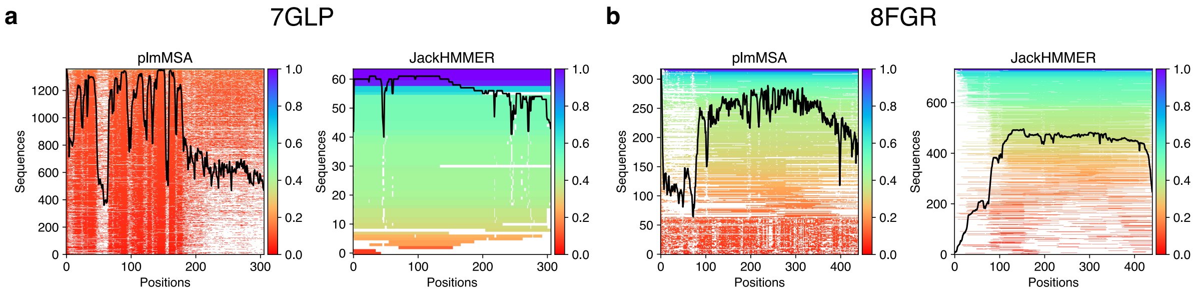

Figure S5: **Comparison of alignment depth obtained with plmMSA and JackHMMER for three complexes in which accurate monomer structures did not translate into correct quaternary assemblies.** **(a)** 7GLP: plmMSA finds $> 10\times$ more sequences than JackHMMER, but many originate from partial or low-identity fragments. **(b)** 8FGR: plmMSA attains the greatest depth yet includes substantial insertion noise, which was removed in the manually cropped MSA that rescued the DockQ score. (See main text.)

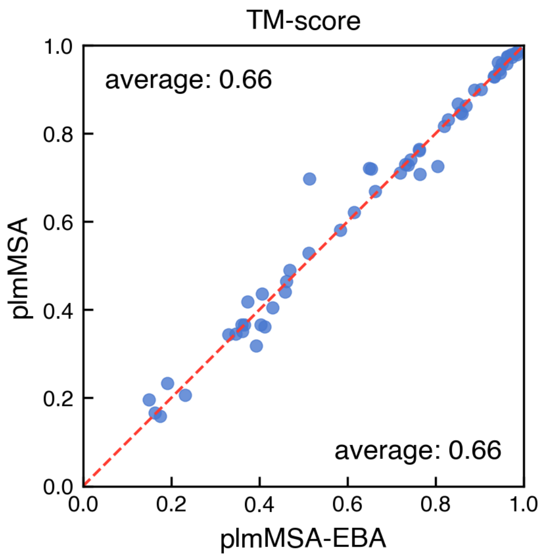

Figure S6: **Comparison of PLMAlign and EBA alignments.** Scatter plot comparing TM-scores obtained using the plmMSA pipeline with PLMAlign (y-axis) versus an alternative pipeline with EBA (x-axis) across CASP15 monomer targets. Each point represents a single target, and the red dashed diagonal indicates parity between the two methods. Both approaches yield an identical average TM-score of 0.66. For detailed per-target plmMSA-EBA results, see Table S11.

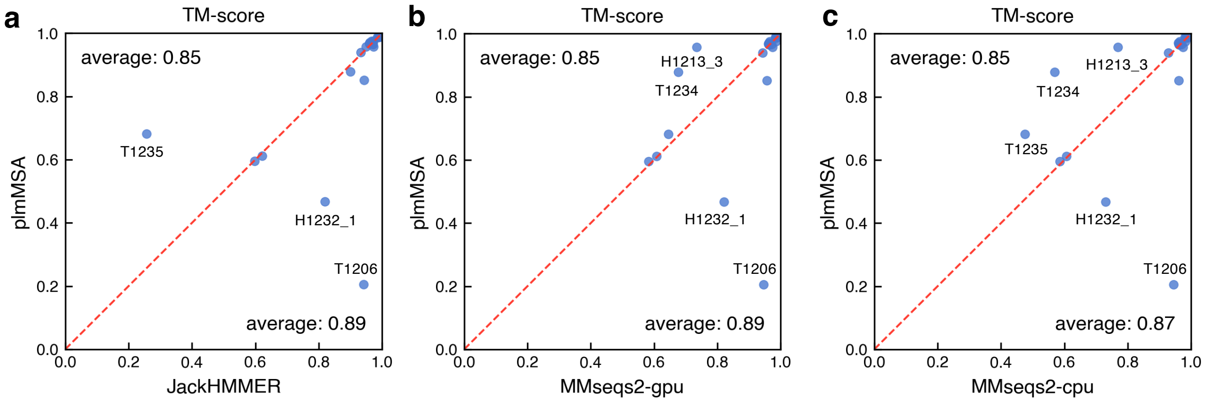

Figure S7: **Benchmarking plmMSA on CASP16 targets.** Scatter plots comparing TM-scores from plmMSA (y-axis) against (a) JackHMMER, (b) MMseqs2-gpu, and (c) MMseqs2-cpu (x-axis) for CASP16 monomer targets. The red dashed line indicates parity. Average TM-scores are reported in each panel, showing comparable or improved performance of plmMSA relative to conventional search methods. Representative outliers (e.g., T1206, T1234, T1235, H1213_3, H1232_1) are labeled. The low accuracy of T1206 and H1232_1 was due to the inclusion of many false positive sequences in the MSA of plmMSA. This suggests that the sequence filtering method needs to be improved. Detailed results are provided in Supplementary Table S12.

S3 Supplementary Tables

Table S1: List of CASP15 benchmark targets used for evaluating monomer structure predictions. The table lists each target along with its corresponding sequence length and the associated Protein Data Bank (PDB) identifier.

Target	Sequence Length	PDB	CASP Classification
T1104	117	7ROA	FM
T1106s1	122	7QIH	FM
T1106s2	114	7QIH	TBM
T1112	460	8ORK	FM
T1113	193	7UYX	FM
T1114s1	189	7UTD	FM
T1114s2	369	7UTD	TBM
T1114s3	535	7UTD	TBM
T1119	153	7SQ4	TBM
T1120	235	7QVB	FM
T1121	381	7TIL	FM
T1122	241	8BBT	FM
T1123	266	7UZT	FM
T1124	384	7UX8	TBM
T1125	1200	8H2N	FM
T1129s2	640	8A8C	FM
T1132	102	8ECX	TBM
T1133	585	8DYS	TBM
T1134s1	230	7UBZ	TBM
T1134s2	313	7UBZ	FM
T1137s1	409	8FEF	FM
T1137s2	343	8FEF	FM
T1137s3	524	8FEF	FM
T1137s4	547	8FEF	TBM
T1137s5	390	8FEF	FM
T1137s6	518	8FEF	FM
T1137s7	653	8FEF	TBM
T1137s8	266	8FEF	TBM
T1137s9	289	8FEF	TBM
T1145	635	7UWW	FM
T1147	103	8EM5	TBM
T1151s2	116	8D5V	FM
T1152	56	7R1L	TBM
T1154	1424	7ZCX	FM
T1155	116	8PBV	FM
T1157s1	1029	8PKO	Not Evaluated
T1157s2	495	8PKO	TBM
T1158	1340	8SXA	TBM
T1159	160	7PZT	FM
T1160	48	8JVN	TBM
T1161	48	8JVP	TBM
T1170	318	7PBR	TBM
T1173	204	8ON4	FM
T1174	338	8OK3	FM
T1176	170	8SMQ	TBM
T1178	306	8UFN	FM
T1179	261	8TN8	FM
T1183	200	8IFX	TBM
T1185s1	350	8OUY	TBM
T1185s2	376	8OUY	TBM
T1185s4	280	8OUY	TBM
T1187	166	8AD2	FM
T1188	630	8C6Z	TBM
T1189	173	7YR7	Not Evaluated
T1190	173	7YR6	Not Evaluated
T1194	168	8OKH	FM

Table S2: Performance evaluation of JackHMMER on CASP15 targets. Metrics include query sequence length, TM-score, RMSD (Å), pLDDT (predicted local distance difference test), *Neff* (effective number of sequences), execution time (seconds), number of sequences retrieved, and sequence overlap.

Target	Seq. Length	TM-score	RMSD	pLDDT	Neff	Execution Time	Num. Sequences
T1104	117	0.717	2.930	92.41	2.18	138.06	13
T1106s1	122	0.630	6.047	74.61	2.35	139.56	13
T1106s2	114	0.894	1.501	97.32	5.32	149.30	71
T1112	460	0.827	2.875	96.43	4.78	409.37	91
T1113	193	0.422	9.846	79.21	1.66	180.77	7
T1114s1	189	0.328	40.856	86.95	4.60	167.93	49
T1114s2	369	0.962	1.431	89.98	6.10	370.73	525
T1114s3	535	0.987	1.426	92.80	4.97	470.22	1058
T1119	153	0.954	0.478	92.23	1.28	172.55	4
T1120	235	0.439	15.543	84.48	2.74	179.54	16
T1121	381	0.514	13.043	96.19	6.53	366.72	509
T1122	241	0.404	19.137	56.60	0.00	228.91	2
T1123	266	0.224	20.778	64.20	0.50	256.05	4
T1124	384	0.847	5.305	90.12	9.36	379.65	1908
T1125	1200	0.247	33.054	62.53	0.85	707.53	5
T1129s2	640	0.395	20.984	71.92	2.34	535.42	13
T1132	102	0.962	0.616	96.08	6.35	136.69	233
T1133	585	0.729	4.800	88.21	6.73	515.61	797
T1134s1	230	0.947	1.377	93.56	7.33	366.53	11993
T1134s2	313	0.763	5.904	87.72	5.70	315.36	437
T1137s1	409	0.395	71.004	85.18	7.26	393.55	579
T1137s2	343	0.408	40.178	85.86	9.16	380.15	3240
T1137s3	524	0.380	55.381	74.44	5.96	592.02	920
T1137s4	547	0.347	50.141	78.23	5.77	623.45	1411
T1137s5	390	0.366	47.618	82.01	7.87	407.55	1088
T1137s6	518	0.361	52.701	78.16	7.20	497.21	1388
T1137s7	653	0.824	10.383	70.97	0.56	973.55	165606
T1137s8	266	0.938	1.736	85.30	8.84	284.72	2367
T1137s9	289	0.945	2.270	89.93	8.23	313.33	2101
T1145	635	0.900	3.080	92.75	4.08	539.10	165
T1147	103	0.982	0.485	92.97	5.53	133.58	159
T1151s2	116	0.874	2.471	78.23	2.38	144.33	70
T1152	56	0.854	0.906	82.91	8.40	125.97	4750
T1154	1424	0.293	28.419	61.63	2.45	794.45	13
T1155	116	0.595	7.901	77.63	2.14	141.48	9
T1157s1	1029	0.701	13.737	83.10	2.06	791.92	2724
T1157s2	495	0.652	7.824	89.79	6.62	504.22	10225
T1158	1340	0.755	7.372	81.69	3.92	2283.12	148479
T1159	160	0.981	0.665	97.72	3.63	149.94	29
T1160	48	0.459	7.776	73.54	5.06	115.37	89
T1161	48	0.454	9.533	84.82	3.24	116.86	14
T1170	318	0.839	3.645	93.05	6.99	345.74	2123
T1173	204	0.338	18.468	85.46	2.74	211.05	452
T1174	338	0.944	1.241	89.22	4.80	376.18	1019
T1176	170	0.758	20.496	94.18	2.99	160.53	19
T1178	306	0.140	29.202	53.77	0.50	312.50	3
T1179	261	0.205	24.042	64.46	0.58	227.62	3
T1183	200	0.940	1.725	94.40	5.36	171.50	558
T1185s1	350	0.842	2.077	84.10	7.96	364.66	2453
T1185s2	376	0.619	6.729	85.26	7.75	379.25	2219
T1185s4	280	0.865	2.864	89.62	6.50	296.81	787
T1187	166	0.936	1.629	92.58	8.04	156.30	1148
T1188	630	0.978	1.001	90.30	5.77	570.04	7494
T1189	55	0.709	1.903	94.08	8.51	118.18	1634
T1190	55	0.463	3.397	94.15	8.51	118.61	1634
T1194	168	0.958	1.435	95.85	2.31	154.24	9

Table S3: Performance evaluation of plmMSA on CASP15 targets. Metrics include query sequence length, TM-score, RMSD (Å), pLDDT (predicted local distance difference test), *Neff* (effective number of sequences), execution time (seconds), number of sequences retrieved, and sequence overlap. Sequence overlap represents the proportion of sequences common to the MSA generated using JackHMMER.

Target	Seq. Length	TM-score	RMSD	pLDDT	Neff	Execution Time	Num. Sequences	Overlap
T1104	117	0.721	2.924	93.64	8.68	5.60	833	0.01
T1106s1	122	0.418	11.533	79.76	7.10	6.17	275	0.01
T1106s2	114	0.899	1.444	95.92	8.60	5.56	802	0.01
T1112	460	0.817	3.127	97.61	9.13	6.41	1246	0.06
T1113	193	0.437	7.828	90.51	8.10	6.03	547	0.01
T1114s1	189	0.441	42.167	86.10	9.06	4.62	1097	0.04
T1114s2	369	0.961	1.495	89.75	8.42	4.54	1048	0.44
T1114s3	535	0.985	1.362	94.23	8.64	5.48	1223	0.63
T1119	153	0.958	0.459	94.49	9.83	6.80	1758	0.00
T1120	235	0.729	4.027	92.87	9.64	5.46	1628	0.01
T1121	381	0.529	13.145	96.48	9.29	5.83	1287	0.31
T1122	241	0.344	14.837	77.85	3.46	6.72	22	0.05
T1123	266	0.207	20.445	69.58	6.48	6.48	178	0.01
T1124	384	0.848	5.010	93.87	9.40	5.11	1394	0.61
T1125	1200	0.234	25.118	73.18	9.07	13.71	1026	0.00
T1129s2	640	0.362	20.812	76.92	8.05	8.99	526	0.01
T1132	102	0.973	0.480	95.92	8.88	5.12	1001	0.07
T1133	585	0.730	4.785	91.20	9.07	7.70	1419	0.39
T1134s1	230	0.959	1.158	96.41	9.56	5.60	1601	0.26
T1134s2	313	0.764	5.884	90.59	9.27	5.46	1445	0.18
T1137s1	409	0.367	46.582	86.12	9.40	6.13	1434	0.29
T1137s2	343	0.405	57.265	88.01	9.28	4.83	1311	0.79
T1137s3	524	0.367	53.163	81.52	9.37	6.01	1479	0.25
T1137s4	547	0.346	63.861	82.76	9.37	5.67	1405	0.43
T1137s5	390	0.367	61.206	87.25	9.39	5.26	1397	0.47
T1137s6	518	0.351	52.505	83.83	9.42	6.48	1456	0.38
T1137s7	653	0.928	2.120	78.61	5.98	11.93	374	0.95
T1137s8	266	0.945	1.531	91.79	9.15	6.63	1269	0.97
T1137s9	289	0.937	3.185	94.86	9.19	4.79	1332	0.80
T1145	635	0.727	8.499	90.14	8.74	11.06	890	0.08
T1147	103	0.982	0.472	93.99	8.71	7.54	964	0.13
T1151s2	116	0.901	1.616	82.65	8.85	7.53	1000	0.06
T1152	56	0.864	0.874	85.86	8.71	7.30	946	0.27
T1154	1424	0.196	34.275	64.89	8.09	13.91	850	0.02
T1155	116	0.621	6.869	72.21	8.68	9.15	820	0.00
T1157s1	1029	0.720	13.275	87.12	7.46	14.83	1106	0.94
T1157s2	495	0.669	6.903	93.06	8.68	8.40	1306	1.00
T1158	1340	0.741	7.739	87.95	8.47	17.02	1211	1.00
T1159	160	0.980	0.763	97.69	9.59	9.69	1554	0.02
T1160	48	0.489	7.926	90.59	7.94	8.12	515	0.05
T1161	48	0.697	2.354	85.86	9.60	7.83	1652	0.00
T1170	318	0.832	3.751	94.83	8.44	8.34	1247	0.63
T1173	204	0.319	16.886	68.52	8.80	11.35	898	0.01
T1174	338	0.709	4.353	85.38	9.46	9.88	1402	0.15
T1176	170	0.762	20.488	95.89	9.11	9.69	1116	0.01
T1178	306	0.167	22.300	78.72	7.73	10.83	425	0.00
T1179	261	0.159	24.497	78.16	7.35	10.40	326	0.00
T1183	200	0.974	1.106	94.65	9.36	7.66	1342	0.12
T1185s1	350	0.845	1.687	91.99	8.81	8.02	1194	0.77
T1185s2	376	0.581	9.331	91.59	8.74	6.09	1167	0.80
T1185s4	280	0.867	2.853	93.66	9.26	7.07	1416	0.28
T1187	166	0.930	1.880	94.93	8.89	7.06	1151	0.68
T1188	630	0.979	0.984	92.97	8.77	11.30	1514	0.83
T1189	55	0.711	1.843	96.11	8.56	7.89	1222	0.71
T1190	55	0.464	3.348	95.78	8.56	4.53	1222	0.71
T1194	168	0.975	0.732	95.70	9.04	9.39	1081	0.01

Table S4: Performance evaluation of MMseqs2-gpu on CASP15 targets. Metrics include query sequence length, TM-score, RMSD (Å), pLDDT (predicted local distance difference test), *Neff* (effective number of sequences), execution time (seconds), number of sequences retrieved, and sequence overlap. Sequence overlap represents the proportion of sequences common to the MSA generated using JackHMMER.

Target	Seq. Length	TM-score	RMSD	pLDDT	Neff	Execution Time	Num. Sequences	Overlap
T1104	117	0.663	3.915	92.24	2.46	5.47	13	0.54
T1106s1	122	0.627	5.584	78.87	2.39	5.34	14	0.71
T1106s2	114	0.910	1.322	95.63	2.54	5.61	13	0.69
T1112	460	0.824	2.939	96.41	4.75	9.31	85	0.94
T1113	193	0.351	14.581	76.56	1.66	9.86	7	0.86
T1114s1	189	0.345	41.007	87.44	4.25	10.10	49	0.88
T1114s2	369	0.956	1.564	90.54	5.47	9.57	296	0.79
T1114s3	535	0.987	0.964	94.58	6.21	10.01	301	0.73
T1119	153	0.948	0.507	91.69	0.00	9.56	2	0.50
T1120	235	0.441	15.922	87.21	2.70	10.00	15	1.00
T1121	381	0.512	11.890	96.93	5.37	9.50	290	0.78
T1122	241	0.442	14.344	60.65	0.00	10.25	2	1.00
T1123	266	0.199	18.024	47.44	0.00	9.74	3	1.00
T1124	384	0.856	4.594	92.78	6.61	9.76	301	0.79
T1125	1200	0.244	31.529	57.14	0.38	9.82	5	1.00
T1129s2	640	0.195	30.269	68.39	2.11	10.03	13	0.92
T1132	102	0.963	0.613	94.69	4.60	5.51	108	0.77
T1133	585	0.730	4.780	91.43	6.21	9.73	301	0.76
T1134s1	230	0.966	0.987	95.99	5.82	9.62	217	0.69
T1134s2	313	0.763	5.889	90.34	5.01	9.42	272	0.86
T1137s1	409	0.347	71.498	85.07	6.29	9.21	301	0.69
T1137s2	343	0.394	50.086	86.72	6.64	9.38	301	0.75
T1137s3	524	0.370	56.829	79.40	3.48	10.21	301	0.70
T1137s4	547	0.347	67.113	79.14	4.25	9.18	301	0.72
T1137s5	390	0.375	66.402	84.22	6.50	9.48	301	0.79
T1137s6	518	0.379	75.309	81.30	5.65	9.83	301	0.72
T1137s7	653	0.927	2.004	77.85	1.41	9.45	301	0.77
T1137s8	266	0.943	1.576	93.88	5.43	8.64	301	0.74
T1137s9	289	0.945	1.563	95.29	5.77	11.60	301	0.77
T1145	635	0.810	7.589	92.40	3.64	10.06	131	0.84
T1147	103	0.981	0.496	93.65	5.31	5.64	143	0.68
T1151s2	116	0.673	3.617	73.96	1.83	5.33	72	0.74
T1152	56	0.872	0.845	83.72	3.81	5.59	301	0.82
T1154	1424	0.176	41.550	58.65	2.40	9.84	14	0.93
T1155	116	0.596	8.030	77.93	1.97	5.19	9	1.00
T1157s1	1029	0.726	13.026	87.33	3.77	10.19	301	0.81
T1157s2	495	0.638	8.187	93.38	6.49	10.07	301	0.76
T1158	1340	0.720	8.093	89.50	6.21	10.89	301	0.72
T1159	160	0.981	0.675	97.84	3.58	9.45	28	0.86
T1160	48	0.302	8.771	67.11	0.58	5.80	3	1.00
T1161	48	0.359	11.680	57.69	0.00	5.69	2	0.50
T1170	318	0.812	4.095	95.30	5.93	9.46	301	0.68
T1173	204	0.374	13.052	84.89	1.43	10.07	23	0.78
T1174	338	0.823	2.432	83.09	2.02	9.53	189	0.78
T1176	170	0.762	20.488	92.64	2.67	9.66	18	0.72
T1178	306	0.155	23.795	58.50	0.32	10.32	4	0.25
T1179	261	0.166	22.243	67.33	0.74	9.47	4	0.75
T1183	200	0.951	1.430	95.31	3.11	9.79	236	0.85
T1185s1	350	0.847	2.052	88.72	5.70	9.73	301	0.69
T1185s2	376	0.662	4.950	91.58	6.11	9.89	301	0.68
T1185s4	280	0.866	2.853	90.05	5.55	10.24	266	0.76
T1187	166	0.934	1.611	93.23	6.69	9.03	301	0.77
T1188	630	0.977	1.036	91.86	4.55	9.75	301	0.72
T1189	55	0.699	1.850	93.75	4.94	5.70	301	0.53
T1190	55	0.449	3.579	93.59	4.94	5.45	301	0.53
T1194	168	0.959	1.407	95.28	1.32	9.38	5	1.00

Table S5: Performance evaluation of MMseqs2-cpu on CASP15 targets. Metrics include query sequence length, TM-score, RMSD (Å), pLDDT (predicted local distance difference test), *Neff* (effective number of sequences), execution time (seconds), number of sequences retrieved, and sequence overlap. Sequence overlap represents the proportion of sequences common to the MSA generated using JackHMMER.

Target	Seq. Length	TM-score	RMSD	pLDDT	Neff	Execution Time	Num. Sequences	Overlap
T1104	117	0.674	3.769	87.65	2.17	181.30	11	0.55
T1106s1	122	0.632	6.559	76.46	2.20	183.68	12	0.75
T1106s2	114	0.919	1.243	95.47	2.12	177.17	10	0.60
T1112	460	0.825	2.896	96.23	4.69	208.23	82	0.94
T1113	193	0.367	14.873	73.07	1.12	177.96	5	0.80
T1114s1	189	0.246	39.265	87.77	3.99	181.06	41	0.90
T1114s2	369	0.957	1.549	90.12	5.61	181.94	295	0.81
T1114s3	535	0.987	0.964	94.55	6.20	177.78	301	0.73
T1119	153	0.949	0.503	91.73	0.00	175.61	2	0.50
T1120	235	0.441	16.264	85.34	2.22	175.88	10	1.00
T1121	381	0.509	12.168	96.95	5.44	177.86	284	0.77
T1122	241	0.286	22.399	65.55	0.00	175.86	2	1.00
T1123	266	0.232	19.866	51.29	0.00	174.09	3	1.00
T1124	384	0.858	4.496	93.51	6.39	183.13	266	0.82
T1125	1200	0.230	32.681	54.20	0.38	176.58	5	1.00
T1129s2	640	0.219	46.067	74.65	2.11	177.18	13	0.92
T1132	102	0.956	0.692	95.42	4.54	180.54	104	0.76
T1133	585	0.731	4.767	90.34	6.21	173.90	301	0.77
T1134s1	230	0.967	0.988	96.06	5.50	176.14	181	0.67
T1134s2	313	0.763	5.891	90.28	5.05	172.64	275	0.86
T1137s1	409	0.347	69.384	84.93	6.22	201.68	301	0.70
T1137s2	343	0.390	67.388	86.80	6.58	176.49	301	0.76
T1137s3	524	0.370	55.610	79.25	3.73	174.09	292	0.72
T1137s4	547	0.345	67.196	78.09	4.23	174.74	301	0.72
T1137s5	390	0.375	67.630	84.34	6.28	194.23	294	0.79
T1137s6	518	0.380	72.979	80.46	5.59	172.37	299	0.73
T1137s7	653	0.930	1.915	77.91	1.40	177.02	301	0.78
T1137s8	266	0.944	1.555	93.25	5.37	173.25	301	0.75
T1137s9	289	0.947	1.550	95.39	5.88	174.87	301	0.76
T1145	635	0.808	8.603	92.14	3.64	176.55	119	0.76
T1147	103	0.981	0.496	93.42	5.08	176.35	119	0.67
T1151s2	116	0.792	4.004	73.11	1.83	180.12	72	0.74
T1152	56	0.870	0.856	84.38	3.69	184.94	301	0.82
T1154	1424	0.248	33.529	64.21	2.41	176.05	15	0.87
T1155	116	0.605	8.589	77.57	1.97	176.63	9	1.00
T1157s1	1029	0.723	12.938	86.99	3.63	175.32	301	0.79
T1157s2	495	0.629	8.519	93.47	6.49	174.12	301	0.76
T1158	1340	0.705	8.589	89.33	5.57	178.61	301	0.67
T1159	160	0.981	0.678	97.98	3.58	177.47	28	0.86
T1160	48	0.415	8.981	73.32	0.00	176.83	2	1.00
T1161	48	0.394	14.069	60.90	0.00	181.08	2	0.50
T1170	318	0.814	4.085	95.01	5.87	177.72	301	0.68
T1173	204	0.403	14.001	85.51	1.42	172.34	16	0.88
T1174	338	0.780	4.174	81.08	2.04	187.34	173	0.76
T1176	170	0.744	20.612	90.77	2.13	179.03	14	0.71
T1178	306	0.154	29.683	55.99	0.32	178.19	4	0.25
T1179	261	0.154	24.534	59.97	0.74	172.80	4	0.75
T1183	200	0.953	1.402	94.48	3.06	171.85	212	0.85
T1185s1	350	0.848	2.026	86.80	5.69	176.21	301	0.70
T1185s2	376	0.622	5.672	91.18	6.10	180.77	301	0.68
T1185s4	280	0.866	2.854	91.90	5.35	172.86	240	0.72
T1187	166	0.939	1.511	93.91	6.67	177.94	301	0.78
T1188	630	0.977	1.057	92.09	4.86	173.04	301	0.71
T1189	55	0.695	1.886	94.46	4.90	180.10	301	0.54
T1190	55	0.451	3.537	94.49	4.90	179.94	301	0.54
T1194	168	0.905	2.127	88.11	0.00	180.21	2	1.00

Table S6: Performance evaluation of plmMSA-Ankh contrastive on CASP15 targets. Metrics include query sequence length, TM-score, RMSD (Å), pLDDT (predicted local distance difference test), *Neff* (effective number of sequences), number of sequences retrieved, and sequence overlap. Sequence overlap represents the proportion of sequences common to the MSA generated using plmMSA-ESM.

Target	Seq. Length	TM-score	RMSD	pLDDT	Neff	Num. Sequences	Overlap
T1104	117	0.728	2.860	93.79	8.90	975	0.47
T1106s1	122	0.343	10.790	84.15	6.56	190	0.05
T1106s2	114	0.895	1.459	94.73	8.90	972	0.17
T1112	460	0.815	3.151	97.74	8.74	956	0.43
T1113	193	0.357	12.659	89.22	7.54	371	0.07
T1114s1	189	0.422	40.995	86.69	8.94	1000	0.78
T1114s2	369	0.961	1.496	89.08	8.35	934	0.89
T1114s3	535	0.986	1.254	94.62	8.47	999	0.70
T1119	153	0.960	0.450	93.41	8.95	1000	0.16
T1120	235	0.724	4.117	93.73	8.90	982	0.18
T1121	381	0.515	12.726	96.56	8.94	1001	0.71
T1122	241	0.411	14.331	76.56	3.39	21	0.14
T1123	266	0.214	21.664	60.57	5.83	114	0.10
T1124	384	0.861	3.323	93.78	8.92	1001	0.61
T1125	1200	0.163	33.325	74.67	8.72	843	0.08
T1129s2	640	0.257	24.677	74.28	7.37	330	0.18
T1132	102	0.975	0.453	95.65	8.85	1001	0.48
T1133	585	0.730	4.793	90.46	8.40	900	0.44
T1134s1	230	0.956	1.226	96.59	8.87	1001	0.38
T1134s2	313	0.761	5.976	90.79	8.83	1001	0.54
T1137s1	409	0.380	73.830	84.77	8.87	1000	0.57
T1137s2	343	0.371	66.667	88.68	8.89	1001	0.69
T1137s3	524	0.366	56.705	80.15	8.80	994	0.52
T1137s4	547	0.348	65.697	80.07	8.89	995	0.59
T1137s5	390	0.364	63.447	86.04	8.91	1001	0.60
T1137s6	518	0.350	51.762	84.31	8.90	993	0.54
T1137s7	653	0.927	2.051	78.06	5.87	365	0.02
T1137s8	266	0.943	1.582	92.04	8.88	1001	0.73
T1137s9	289	0.949	2.261	94.68	8.87	1001	0.65
T1145	635	0.762	10.619	91.42	8.24	638	0.11
T1151s2	116	0.915	1.408	79.43	8.76	1000	0.52
T1154	1424	0.187	39.451	62.94	7.86	726	0.17
T1158	1340	0.723	8.179	90.31	8.44	1001	0.12
T1159	160	0.983	0.623	97.83	8.95	993	0.20
T1170	318	0.845	3.430	94.61	7.98	996	0.59
T1173	204	0.325	16.115	72.12	8.29	629	0.16
T1178	306	0.175	22.885	79.72	7.58	383	0.06
T1179	261	0.161	25.833	78.19	6.69	207	0.12
T1185s1	350	0.856	1.570	91.51	8.69	1001	0.40
T1185s2	376	0.581	9.361	91.58	8.62	1000	0.32
T1185s4	280	0.867	2.857	92.60	8.82	1001	0.58
T1187	166	0.927	1.899	95.04	8.71	983	0.82
T1188	630	0.980	0.952	93.17	8.07	995	0.43
T1194	168	0.982	0.537	95.48	8.88	972	0.01

Table S7: Performance evaluation of plmMSA-ESM on CASP15 targets. Metrics include TM-score, RMSD (Å), pLDDT (predicted local distance difference test), *Neff* (effective number of sequences), number of sequences retrieved, and sequence overlap. Sequence overlap represents the proportion of sequences common to the MSA generated using plmMSA-Ankh.

Target	Seq. Length	TM-score	RMSD	pLDDT	Neff	Num. Sequences	Overlap
T1104	117	0.719	2.937	93.56	8.68	833	0.55
T1106s1	122	0.327	13.640	78.77	7.10	275	0.03
T1106s2	114	0.897	1.467	95.76	8.60	802	0.20
T1112	460	0.819	3.068	97.25	8.33	698	0.58
T1113	193	0.390	14.019	78.35	6.65	201	0.12
T1114s1	189	0.365	42.618	87.20	8.74	874	0.89
T1114s2	369	0.963	1.405	89.07	8.30	941	0.88
T1114s3	535	0.984	1.418	95.34	8.17	919	0.76
T1119	153	0.957	0.469	94.26	8.96	920	0.18
T1120	235	0.564	7.485	92.44	8.67	821	0.21
T1121	381	0.530	12.883	96.40	8.92	1001	0.71
T1122	241	0.262	19.246	68.29	1.00	4	0.75
T1123	266	0.212	23.075	71.31	5.23	75	0.15
T1124	384	0.861	3.211	93.34	8.93	1001	0.61
T1125	1200	0.165	34.013	73.46	7.10	248	0.26
T1129s2	640	0.239	27.909	74.10	7.03	257	0.24
T1132	102	0.972	0.489	95.83	8.88	1001	0.48
T1133	585	0.730	4.778	91.44	8.41	919	0.44
T1134s1	230	0.961	1.122	96.14	8.85	985	0.39
T1134s2	313	0.764	5.874	90.39	8.66	989	0.55
T1137s1	409	0.366	60.796	83.71	8.91	1000	0.57
T1137s2	343	0.398	66.490	88.09	8.89	1000	0.69
T1137s3	524	0.392	56.353	81.07	8.87	1000	0.52
T1137s4	547	0.348	56.050	81.09	8.89	1000	0.59
T1137s5	390	0.368	63.833	86.77	8.91	1000	0.60
T1137s6	518	0.350	63.306	82.12	8.86	1000	0.54
T1137s7	653	0.801	10.960	71.96	2.91	15	0.40
T1137s8	266	0.943	1.556	91.31	8.78	1001	0.73
T1137s9	289	0.945	2.271	94.96	8.70	986	0.66
T1145	635	0.776	9.389	90.51	7.33	324	0.22
T1151s2	116	0.910	1.449	81.29	8.85	1000	0.52
T1154	1424	0.177	35.798	60.22	6.35	248	0.50
T1158	1340	0.756	7.327	89.80	4.65	327	0.36
T1159	160	0.966	1.003	96.64	8.56	760	0.26
T1170	318	0.830	3.760	94.80	7.92	834	0.70
T1173	204	0.342	18.421	81.87	7.52	369	0.27
T1178	306	0.169	22.640	80.87	5.04	66	0.36
T1179	261	0.158	24.026	75.34	6.16	143	0.17
T1185s1	350	0.845	1.749	92.16	7.50	593	0.67
T1185s2	376	0.629	5.391	92.26	7.21	489	0.66
T1185s4	280	0.867	2.856	91.95	8.71	996	0.58
T1187	166	0.934	1.809	95.45	8.65	976	0.83
T1188	630	0.978	1.002	93.34	8.34	947	0.45
T1194	168	0.802	2.915	92.45	5.88	118	0.08

Table S8: Detailed execution time breakdown of the plmMSA module for CASP15 targets. The table reports the processing time (in seconds) for each major stage of the workflow: vector database (VDB) search, embedding loading, and PLMAlign computation.

Target	Seq. Length	VDB Search (s)	Embedding Load (s)	PLMAlign (s)
T1104	117	0.91	2.35	2.30
T1106s1	122	1.13	2.11	2.88
T1106s2	114	1.04	1.91	2.56
T1112	460	0.90	2.16	3.26
T1113	193	1.04	2.22	2.71
T1114s1	189	0.99	1.85	1.75
T1114s2	369	0.88	1.51	2.08
T1114s3	535	0.88	1.58	2.94
T1119	153	0.86	2.47	3.38
T1120	235	0.81	1.88	2.70
T1121	381	1.11	1.88	2.78
T1122	241	0.98	2.33	3.34
T1123	266	0.98	2.27	3.15
T1124	384	1.00	1.66	2.39
T1125	1200	3.15	3.16	7.17
T1129s2	640	0.98	3.09	4.79
T1132	102	1.07	1.62	2.40
T1133	585	1.06	2.26	4.25
T1134s1	230	1.03	1.70	2.81
T1134s2	313	0.85	1.70	2.85
T1137s1	409	0.90	2.42	2.74
T1137s2	343	0.83	1.62	2.33
T1137s3	524	0.80	1.87	3.24
T1137s4	547	0.77	1.63	3.18
T1137s5	390	0.96	1.66	2.57
T1137s6	518	0.89	2.33	3.18
T1137s7	653	1.24	6.02	4.55
T1137s8	266	1.31	3.41	1.86
T1137s9	289	0.93	1.69	2.12
T1145	635	1.29	5.45	4.19
T1147	103	0.98	4.21	2.31
T1151s2	116	1.08	4.10	2.31
T1152	56	1.10	3.97	2.19
T1154	1424	3.28	4.70	5.76
T1155	116	1.09	5.25	2.77
T1157s1	1029	3.48	4.66	6.52
T1157s2	495	1.25	4.19	2.89
T1158	1340	3.22	6.47	7.08
T1159	160	1.09	5.43	3.10
T1160	48	0.99	4.77	2.31
T1161	48	0.96	4.11	2.73
T1170	318	1.25	4.51	2.52
T1173	204	1.14	6.86	3.28
T1174	338	1.22	4.71	3.85
T1176	170	1.15	5.25	3.22
T1178	306	1.09	5.97	3.68
T1179	261	1.16	5.80	3.37
T1183	200	1.33	4.09	2.20
T1185s1	350	1.08	4.25	2.60
T1185s2	376	1.10	2.09	2.82
T1185s4	280	1.14	3.10	2.77
T1187	166	1.38	3.86	1.75
T1188	630	1.44	5.62	4.10
T1189	55	1.02	4.86	1.98
T1190	55	0.83	1.47	2.21
T1194	168	1.06	5.28	2.97

Table S9: Evaluation of structure prediction accuracy on protein complex targets.

Structure				plmMSA only		DeepFold-PLM		AF2-Multimer	
PDB ID	Oligo. State	Stoich.	Length	TMscore	DockQ	TMscore	DockQ	TMscore	DockQ
5SNJ	Homo 2-mer	A2	828	0.996	0.943	0.997	0.926	0.996	0.933
7CJS	Homo 4-mer	A4	1016	0.987	0.800	0.988	0.805	0.987	0.806
7F13	Homo 2-mer	A2	344	0.966	0.539	0.936	0.523	0.973	0.596
7F8S	Homo 2-mer	A2	466	0.505	0.010	0.506	0.010	0.507	0.010
7GLP	Homo 2-mer	A2	612	0.327	0.030	0.330	0.018	0.838	0.345
7KO3	Homo 2-mer	A2	620	0.983	0.885	0.989	0.917	0.988	0.949
7MNY	Hetero 2-mer	A1B1	358	0.974	0.784	0.977	0.784	0.969	0.790
7NZO	Homo 2-mer	A2	408	0.994	0.936	0.994	0.929	0.994	0.933
7OJR	Homo 2-mer	A2	928	0.924	0.766	0.910	0.704	0.922	0.732
7OQK	Homo 2-mer	A2	514	0.995	0.917	0.993	0.869	0.995	0.916
7Q6J	Homo 2-mer	A2	560	0.499	0.046	0.511	0.037	0.504	0.025
7QRR	Homo 3-mer	A3	459	0.240	0.053	0.378	0.049	0.240	0.038
7R2O	Homo 2-mer	A2	958	0.989	0.851	0.993	0.835	0.995	0.837
7RUV	Homo 3-mer	A3	588	0.976	0.864	0.976	0.873	0.976	0.882
7SR4	Hetero 2-mer	A1B1	545	0.763	0.041	0.773	0.036	0.780	0.032
7SZG	Homo 3-mer	A3	1002	0.990	0.930	0.991	0.927	0.991	0.925
7T0Q	Homo 2-mer	A2	496	0.990	0.906	0.991	0.907	0.989	0.911
7TOT	Homo 2-mer	A2	588	0.897	0.868	0.889	0.838	0.894	0.872
7V1Q	Homo 4-mer	A4	1004	0.997	0.960	0.995	0.918	0.997	0.956
7V5V	Homo 2-mer	A2	416	0.980	0.882	0.979	0.867	0.973	0.871
7VO5	Homo 2-mer	A2	572	0.951	0.836	0.946	0.815	0.955	0.824
7X6H	Homo 2-mer	A2	350	0.937	0.821	0.945	0.836	0.944	0.829
7XKY	Hetero 2-mer	A1B1	493	0.958	0.805	0.973	0.840	0.954	0.786
7XMK	Homo 2-mer	A2	588	0.491	0.017	0.495	0.016	0.489	0.006
8A3O	Homo 2-mer	A2	712	0.981	0.787	0.981	0.798	0.974	0.790
8AI3	Homo 2-mer	A2	688	0.961	0.790	0.962	0.787	0.972	0.842
8BEM	Homo 2-mer	A2	606	0.485	0.005	0.487	0.005	0.500	0.009
8BLJ	Homo 2-mer	A2	564	0.514	0.016	0.901	0.544	0.503	0.012
8BQO	Hetero 4-mer	A2B2	642	0.993	0.955	0.992	0.949	0.992	0.950
8BXX	Homo 2-mer	A2	772	0.993	0.933	0.993	0.920	0.994	0.934
8DEN	Homo 2-mer	A2	212	0.525	0.063	0.550	0.137	0.529	0.067
8EK4	Homo 2-mer	A2	282	0.505	0.006	0.528	0.031	0.347	0.014
8EN9	Homo 3-mer	A3	942	0.996	0.959	0.992	0.905	0.991	0.954
8FGR	Homo 2-mer	A2	880	0.957	0.494	0.939	0.437	0.994	0.922
8GBK	Homo 7-mer	A7	721	0.327	0.132	0.269	0.045	0.339	0.090
8GLE	Hetero 2-mer	A1B1	401	0.962	0.820	0.973	0.838	0.966	0.829
8HCI	Hetero 2-mer	A1B1	573	0.930	0.528	0.916	0.504	0.938	0.566
8JBP	Homo 2-mer	A2	542	0.991	0.903	0.993	0.918	0.989	0.900
8JEL	Hetero 3-mer	A1B1C1	551	0.808	0.319	0.817	0.328	0.820	0.324
8JIK	Homo 2-mer	A2	842	0.990	0.755	0.995	0.895	0.996	0.911
8JT0	Homo 2-mer	A2	406	0.983	0.701	0.981	0.689	0.977	0.700
8K4R	Hetero 2-mer	A1B1	642	0.888	0.494	0.884	0.505	0.890	0.557
8KE1	Homo 2-mer	A2	554	0.988	0.935	0.972	0.821	0.984	0.904
8OYL	Homo 4-mer	A4	128	0.904	0.836	0.915	0.833	0.465	0.352
8PRQ	Homo 4-mer	A4	336	0.995	0.933	0.996	0.932	0.994	0.943
8Q6M	Homo 2-mer	A2	308	0.988	0.898	0.990	0.903	0.989	0.902
8S9K	Homo 2-mer	A2	560	0.965	0.572	0.975	0.696	0.972	0.673
8X6P	Homo 2-mer	A2	216	0.557	0.063	0.690	0.330	0.716	0.319
8YSH	Hetero 2-mer	A1B1	325	0.417	0.043	0.407	0.075	0.426	0.021
9AVA	Homo 2-mer	A2	462	0.982	0.882	0.988	0.867	0.988	0.910

Table S10: End-to-end computational times (in seconds) for DeepFold-PLM (DF-PLM) and AF2-Multimer (AF2-M) across target complexes.

			MSA		Prediction		Total	
Target	Stoich.	Length	DF-PLM	AF2-M	DF-PLM	AF2-M	DF-PLM	AF2-M
8OYL	A4	128	2.4	30.2	135.9	54.0	32.6	189.9
8DEN	A2	212	9.7	41.0	246.6	41.8	50.7	288.5
8X6P	A2	216	3.5	41.6	182.2	41.7	45.1	223.9
8EK4	A2	282	10.6	52.4	219.2	43.7	63.0	263.0
8Q6M	A2	308	6.7	57.3	188.7	47.1	64.0	235.9
8YSH	A1B1	325	9.0	60.7	210.8	50.1	69.7	260.9
8PRQ	A4	336	6.7	63.0	216.0	52.4	69.6	268.4
7F13	A2	344	5.3	64.7	223.7	54.2	69.9	277.9
7X6H	A2	350	2.6	66.0	212.1	55.6	68.6	267.8
7MNY	A1B1	358	5.3	67.8	179.4	57.7	73.1	237.1
8GLE	A1B1	401	6.7	78.1	239.2	70.8	84.8	310.0
8JT0	A2	406	3.1	79.4	156.4	72.6	82.4	229.0
7NZO	A2	408	5.4	79.9	170.7	73.3	85.3	244.0
7V5V	A2	416	4.9	82.0	231.5	76.3	86.9	307.7
7QRR	A3	459	3.0	94.1	163.8	94.4	97.2	258.2
9AVA	A2	462	6.7	95.0	208.6	95.8	101.7	304.4
7F8S	A2	466	6.8	96.2	210.9	97.7	103.1	308.6
7XKY	A1B1	493	8.2	104.7	170.1	111.3	112.9	281.4
7T0Q	A2	496	12.7	105.7	180.2	112.8	118.4	293.0
7OQK	A2	514	8.7	111.7	210.1	122.8	120.5	332.9
8JBP	A2	542	4.8	121.7	196.3	139.4	126.5	335.7
7SR4	A1B1	545	6.3	122.8	189.0	141.2	129.1	330.3
8JEL	A1B1C1	551	1.0	125.1	247.7	145.0	126.1	392.7
8KE1	A2	554	5.9	126.2	233.5	146.9	132.1	380.4
8S9K	A2	560	9.4	128.5	196.6	150.8	137.9	347.4
7Q6J	A2	560	9.8	128.5	270.1	150.8	138.3	420.9
8BLJ	A2	564	8.2	130.0	209.8	153.4	138.2	363.2
7VO5	A2	572	12.3	133.2	207.6	158.8	145.5	366.4
8HCI	A1B1	573	3.8	133.6	248.4	159.5	137.4	407.9
7XMK	A2	588	9.8	139.6	183.2	169.8	149.4	353.0
7RUV	A3	588	12.5	139.6	274.7	169.8	152.2	444.5
7TOT	A2	588	0.3	139.6	143.7	169.8	139.9	313.5
8BEM	A2	606	9.0	147.2	143.0	182.7	156.2	325.7
7GLP	A2	612	8.4	149.8	188.0	187.2	158.2	375.1
7KO3	A2	620	12.8	153.3	307.9	193.2	166.0	501.1
8BQO	A2B2	642	3.0	163.3	194.9	210.3	166.3	405.2
8K4R	A1B1	642	4.5	163.3	220.6	210.3	167.8	430.9
8AI3	A2	688	5.0	185.8	209.5	248.7	190.8	458.2
8A3O	A2	712	5.8	198.5	229.0	270.2	204.2	499.1
8GBK	A7	721	4.2	203.4	245.2	278.5	207.6	523.7
8BXX	A2	772	6.1	233.0	249.0	327.9	239.2	576.9
5SNJ	A2	828	3.1	269.3	213.4	386.9	272.4	600.3
8JIK	A2	842	4.2	278.9	249.9	402.3	283.1	652.3
8FGR	A2	880	9.5	306.5	209.1	445.8	316.1	654.9
7OJR	A2	928	2.2	344.2	215.3	503.6	346.4	719.0
8EN9	A3	942	5.9	355.8	203.1	521.1	361.7	724.3
7R2O	A2	958	7.6	369.4	259.4	541.4	377.0	800.8
7SZG	A3	1002	10.3	408.8	183.1	599.0	419.1	782.1
7V1Q	A4	1004	7.8	410.7	196.6	601.7	418.5	798.3
7CJS	A4	1016	9.2	422.0	161.1	617.8	431.2	778.9

Table S11: Performance evaluation of EBA on CASP15 targets. Metrics include query sequence length, TM-score, RMSD (Å), and pLDDT (predicted local distance difference test).

Target	Seq. Length	TM-score	RMSD	pLDDT
T1104	117	0.649	3.994	81.58
T1106s1	122	0.374	10.932	83.06
T1106s2	114	0.887	1.502	95.26
T1112	460	0.819	3.072	97.28
T1113	193	0.406	10.277	90.47
T1114s1	189	0.458	40.376	86.64
T1114s2	369	0.941	1.908	90.03
T1114s3	535	0.985	1.346	95.33
T1119	153	0.950	0.519	95.28
T1120	235	0.738	4.025	92.95
T1121	381	0.511	13.617	96.21
T1122	241	0.330	19.224	68.69
T1123	266	0.232	16.249	65.07
T1124	384	0.857	3.120	93.20
T1125	1200	0.191	31.506	72.93
T1129s2	640	0.412	17.535	70.56
T1132	102	0.972	0.487	95.55
T1133	585	0.731	4.777	90.93
T1134s1	230	0.961	1.123	96.29
T1134s2	313	0.763	5.959	91.42
T1137s1	409	0.366	52.892	83.79
T1137s2	343	0.430	65.342	87.51
T1137s3	524	0.403	59.226	80.97
T1137s4	547	0.347	66.057	79.84
T1137s5	390	0.360	62.555	86.03
T1137s6	518	0.361	56.512	84.46
T1137s7	653	0.932	1.991	78.05
T1137s8	266	0.945	1.500	88.40
T1137s9	289	0.946	2.221	93.63
T1145	635	0.805	8.305	92.54
T1147	103	0.981	0.490	94.04
T1151s2	116	0.902	1.583	83.70
T1152	56	0.868	0.854	86.57
T1154	1424	0.149	40.334	62.66
T1155	116	0.614	6.980	74.78
T1157s1	1029	0.654	30.993	84.31
T1157s2	495	0.662	7.048	92.80
T1158	1340	0.743	7.749	85.86
T1159	160	0.985	0.597	97.59
T1160	48	0.469	8.015	91.39
T1161	48	0.514	8.404	84.24
T1170	318	0.828	3.827	93.82
T1173	204	0.393	16.807	73.77
T1174	338	0.764	3.389	89.27
T1176	170	0.762	20.503	96.21
T1178	306	0.162	22.356	74.31
T1179	261	0.175	24.779	69.54
T1183	200	0.964	1.071	95.07
T1185s1	350	0.860	1.512	92.13
T1185s2	376	0.584	9.218	91.32
T1185s4	280	0.850	3.003	90.47
T1187	166	0.932	1.699	94.78
T1188	630	0.972	1.327	92.59
T1189	55	0.719	1.831	95.31
T1190	55	0.462	3.481	95.46
T1194	168	0.963	1.350	96.94

Table S12: Performance evaluation of plmMSA, JackHMMER, MMseqs2-gpu, and MMseqs2-cpu on CASP16 targets. Reported metrics include TM-score, RMSD (Å), and pLDDT. CASP16 targets with strong multimer interactions were excluded to ensure fair comparison of monomeric structure prediction accuracy.

Target (Length)	Method	TM-score	RMSD	pLDDT
T1201 (210)	plmMSA	0.612	9.362	77.08
	JackHMMER	0.621	11.988	80.57
	MMseqs2-gpu	0.609	8.173	79.74
	MMseqs2-cpu	0.607	9.860	78.96
T1206 (237)	plmMSA	0.206	23.816	76.10
	JackHMMER	0.942	1.733	90.37
	MMseqs2-gpu	0.946	1.697	90.21
	MMseqs2-cpu	0.945	1.679	90.37
T1212 (466)	plmMSA	0.852	8.942	89.39
	JackHMMER	0.944	1.996	91.90
	MMseqs2-gpu	0.956	1.771	93.40
	MMseqs2-cpu	0.962	1.645	94.18
T1214 (677)	plmMSA	0.987	1.056	96.08
	JackHMMER	0.990	0.892	93.01
	MMseqs2-gpu	0.987	1.063	93.21
	MMseqs2-cpu	0.988	1.033	93.15
T1227s1 (427)	plmMSA	0.968	1.345	93.04
	JackHMMER	0.960	1.509	86.29
	MMseqs2-gpu	0.961	1.495	93.29
	MMseqs2-cpu	0.962	1.463	93.23
T1234 (413)	plmMSA	0.878	8.697	89.34
	JackHMMER	0.901	10.777	92.05
	MMseqs2-gpu	0.676	8.351	88.33
	MMseqs2-cpu	0.570	11.842	84.10
T1235 (115)	plmMSA	0.682	4.578	81.02
	JackHMMER	0.257	11.324	67.11
	MMseqs2-gpu	0.645	9.915	80.64
	MMseqs2-cpu	0.475	7.541	79.16
T1266 (336)	plmMSA	0.970	1.145	91.44
	JackHMMER	0.961	1.309	87.57
	MMseqs2-gpu	0.963	1.265	94.57
	MMseqs2-cpu	0.960	1.323	94.49
T1269 (1410)	plmMSA	0.595	55.399	86.84
	JackHMMER	0.598	55.427	82.46
	MMseqs2-gpu	0.583	54.970	81.56
	MMseqs2-cpu	0.586	55.124	82.09
T1280 (237)	plmMSA	0.975	0.964	95.94
	JackHMMER	0.967	1.132	95.40
	MMseqs2-gpu	0.985	0.752	96.53
	MMseqs2-cpu	0.983	0.787	96.12
T1299 (168)	plmMSA	0.967	0.972	95.34
	JackHMMER	0.970	0.930	93.98
	MMseqs2-gpu	0.971	0.904	94.17
	MMseqs2-cpu	0.971	0.906	94.46
H1204_1 (140)	plmMSA	0.957	1.304	90.33
	JackHMMER	0.974	0.719	93.16
	MMseqs2-gpu	0.974	0.705	90.67
	MMseqs2-cpu	0.974	0.708	90.31
H1204_2 (147)	plmMSA	0.965	0.962	97.42
	JackHMMER	0.971	0.900	97.41
	MMseqs2-gpu	0.964	0.972	96.59
	MMseqs2-cpu	0.962	1.005	96.96
H1204_3 (142)	plmMSA	0.975	0.961	97.61
	JackHMMER	0.973	0.970	97.64
	MMseqs2-gpu	0.966	1.026	97.15
	MMseqs2-cpu	0.964	1.057	97.14
H1213_2 (330)	plmMSA	0.989	0.809	92.01
	JackHMMER	0.989	0.788	91.52
	MMseqs2-gpu	0.988	0.847	90.58
	MMseqs2-cpu	0.981	1.034	90.39
H1213_3 (377)	plmMSA	0.957	2.040	92.98
	JackHMMER	0.949	2.233	94.89
	MMseqs2-gpu	0.735	10.795	89.71
	MMseqs2-cpu	0.769	5.996	90.53
H1213_4 (282)	plmMSA	0.988	0.654	96.08
	JackHMMER	0.989	0.638	95.90

Target (Length)	Method	TM-score	RMSD	pLDDT
	MMseqs2-gpu	0.984	0.832	94.75
	MMseqs2-cpu	0.983	0.831	94.69
H1213_5 (323)	plmMSA	0.939	2.305	84.65
	JackHMMER	0.933	2.312	78.73
	MMseqs2-gpu	0.943	1.766	77.06
	MMseqs2-cpu	0.928	2.092	76.74
H1227_1 (427)	plmMSA	0.967	1.362	92.04
	JackHMMER	0.961	1.500	85.61
	MMseqs2-gpu	0.963	1.459	93.48
	MMseqs2-cpu	0.961	1.495	92.60
H1232_1 (220)	plmMSA	0.467	17.983	78.43
	JackHMMER	0.819	4.616	84.34
	MMseqs2-gpu	0.821	4.599	82.06
	MMseqs2-cpu	0.731	6.250	82.61
H1232_2 (242)	plmMSA	0.987	0.656	92.87
	JackHMMER	0.985	0.741	92.27
	MMseqs2-gpu	0.985	0.728	91.90
	MMseqs2-cpu	0.985	0.725	91.96

S4 Evaluating MSA quality for structural accuracy

Figure S8a shows the correlation between MSA quality metrics and structural accuracy (TM-score). Although the number of effective sequences (*Neff*) has been widely used to assess MSA diversity, we observe that Evoformer-based masked MSA loss exhibits a clearer threshold-like relationship with the TM score: high loss values consistently correspond to lower structural accuracy.

These results underscore the limitations of conventional diversity-based metrics like N_{eff} and highlight the value of loss-based neural metrics for assessing alignment quality. Efficient identification of low-quality MSAs can reduce unnecessary inference, particulary for long sequences where computational cost increases rapidly. To address this, we plan to integrate the loss metric into our web service, enabling early feedback on MSA quality before structure prediction begins (Fig. S8b). For broader scalability, future efforts should focus on developing lightweight and fast alternatives that approximate this loss metric without requiring full model inference.

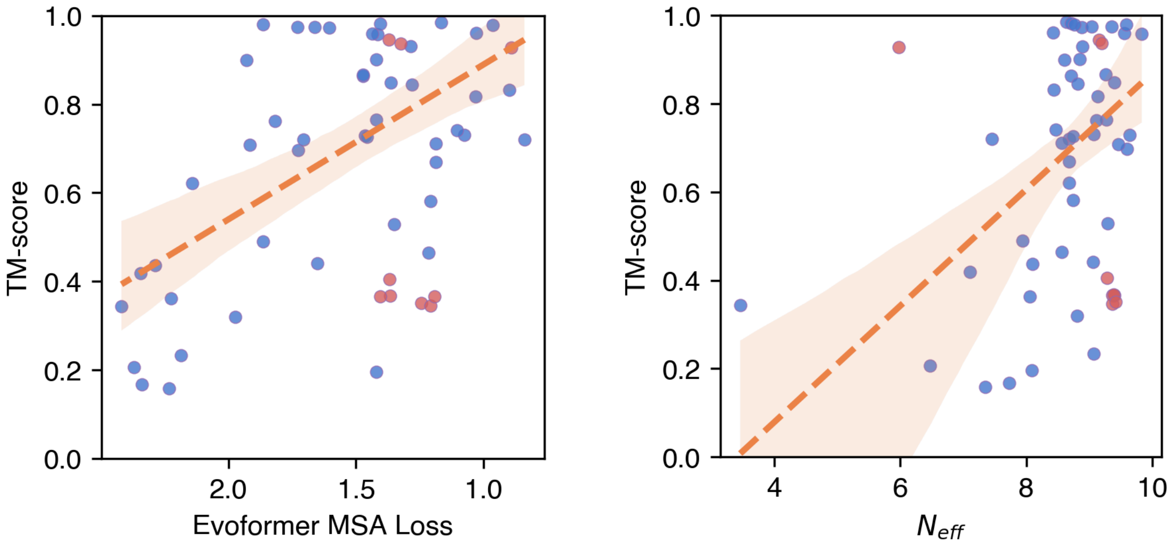

Figure S8: **MSA Quality Assessment.** Comparison between Evoformer MSA Loss (left) and *Neff* metric (right) versus TM-score. The loss-based metric shows a clearer correlation with structure accuracy. Red points indicate cases dominated by inter-chain interactions, where alignment quality alone may be insufficient.
